# Supplementary material for: Changes in Head and Pelvic Movement Symmetry after Diagnostic Anaesthesia: Interactions between Subjective Judgement Categories and Commonly Applied Blocks
Source: Animals (Basel). 2023 Dec 6;13(24):3769. doi: 10.3390/ani13243769 (PMC10740945; doi:10.3390/ani13243769)
Supplement: Supplementary file 1 [file animals-13-03769-s001.zip › animals-2705597-supplementary.pdf]

**Supplementary data**

**Table S1:** Pairwise comparisons for two-way interaction model between forelimb diagnostic anaesthesia 'efficacy' and 'type' for outcome variable DHDmin, i.e. head movement asymmetry associated with changes in weight-bearing between contralateral forelimbs.
Given are mean differences (in mm), standard error (in mm), degrees of freedom, significance achieved, upper and lower bounds of 95% confidence intervals for the pairwise difference (in mm).
Pairwise significant differences at P<0.05 after Bonferroni correction are indicated with '*'.
Forelimb block types: ASNB: abaxial sesamoid nerve block; Low4: Low4-point nerve block; PD: palmar digital nerve block; other: all other nerve/joint blocks.
Efficacy: neg: negative block (0 to 30% perceived change); part pos: partially positive block (>30 to 70% perceived change; pos: positive block (>70% change).

**Pairwise Comparisons[a]**

| (I) Type x Efficacy | (J) Type x Efficacy | Mean Difference (I-J) | Std. Error | df | Sig.[c] | 95% Confidence Interval for Difference[c] | |
|---|---|---|---|---|---|---|---|
| | | | | | | Lower Bound | Upper Bound |
| ASNB_neg | ASNB_part pos | 8.650 | 4.824 | 252.490 | 1.000 | -7.797 | 25.097 |
| | ASNB_pos | 9.857 | 4.970 | 252.999 | 1.000 | -7.087 | 26.800 |
| | Low4_neg | 3.151 | 4.300 | 205.714 | 1.000 | -11.550 | 17.851 |
| | Low4_part pos | 13.263 | 5.254 | 226.078 | .810 | -4.676 | 31.203 |
| | Low4_pos | 25.656* | 4.692 | 249.994 | <0.001 | 9.656 | 41.656 |
| | other_neg | .713 | 3.225 | 218.400 | 1.000 | -10.305 | 11.731 |
| | other_part pos | 3.707 | 3.580 | 244.411 | 1.000 | -8.504 | 15.918 |
| | other_pos | 4.626 | 4.288 | 252.528 | 1.000 | -9.994 | 19.246 |
| | PD_neg | -5.969 | 3.629 | 212.034 | 1.000 | -18.372 | 6.435 |
| | PD_part pos | 5.382 | 4.343 | 250.253 | 1.000 | -9.427 | 20.192 |
| | PD_pos | 10.349 | 3.686 | 248.901 | .355 | -2.220 | 22.917 |
| ASNB_part pos | ASNB_neg | -8.650 | 4.824 | 252.490 | 1.000 | -25.097 | 7.797 |
| | ASNB_pos | 1.207 | 5.596 | 251.722 | 1.000 | -17.873 | 20.286 |
| | Low4_neg | -5.499 | 5.091 | 247.817 | 1.000 | -22.861 | 11.863 |
| | Low4_part pos | 4.614 | 5.874 | 247.844 | 1.000 | -15.418 | 24.645 |
| | Low4_pos | 17.007 | 5.313 | 249.444 | .102 | -1.111 | 35.124 |
| | other_neg | -7.937 | 4.213 | 252.249 | 1.000 | -22.301 | 6.427 |
| | other_part pos | -4.943 | 4.446 | 251.780 | 1.000 | -20.101 | 10.215 |

| | | | | | | | |
|---|---|---|---|---|---|---|---|
| | other_pos | -4.024 | 4.973 | 251.727 | 1.000 | -20.978 | 12.931 |
| | PD_neg | -14.618 | 4.472 | 246.252 | .081 | -29.871 | .634 |
| | PD_part pos | -3.267 | 5.077 | 252.016 | 1.000 | -20.577 | 14.042 |
| | PD_pos | 1.699 | 4.475 | 247.800 | 1.000 | -13.563 | 16.961 |
| ASNB_pos | ASNB_neg | -9.857 | 4.970 | 252.999 | 1.000 | -26.800 | 7.087 |
| | ASNB_part pos | -1.207 | 5.596 | 251.722 | 1.000 | -20.286 | 17.873 |
| | Low4_neg | -6.706 | 5.212 | 249.710 | 1.000 | -24.477 | 11.065 |
| | Low4_part pos | 3.407 | 6.042 | 251.731 | 1.000 | -17.195 | 24.008 |
| | Low4_pos | 15.800 | 5.403 | 250.319 | .249 | -2.624 | 34.224 |
| | other_neg | -9.143 | 4.432 | 244.745 | 1.000 | -24.259 | 5.972 |
| | other_part pos | -6.150 | 4.575 | 252.576 | 1.000 | -21.747 | 9.448 |
| | other_pos | -5.231 | 5.140 | 248.565 | 1.000 | -22.759 | 12.297 |
| | PD_neg | -15.825 | 4.661 | 252.795 | .052 | -31.715 | .064 |
| | PD_part pos | -4.474 | 5.128 | 249.458 | 1.000 | -21.961 | 13.013 |
| | PD_pos | .492 | 4.578 | 252.837 | 1.000 | -15.115 | 16.099 |
| Low4_neg | ASNB_neg | -3.151 | 4.300 | 205.714 | 1.000 | -17.851 | 11.550 |
| | ASNB_part pos | 5.499 | 5.091 | 247.817 | 1.000 | -11.863 | 22.861 |
| | ASNB_pos | 6.706 | 5.212 | 249.710 | 1.000 | -11.065 | 24.477 |
| | Low4_part pos | 10.113 | 5.589 | 235.822 | 1.000 | -8.960 | 29.186 |
| | Low4_pos | 22.506* | 5.027 | 249.013 | **.001** | 5.362 | 39.649 |
| | other_neg | -2.438 | 3.676 | 223.936 | 1.000 | -14.990 | 10.115 |
| | other_part pos | .556 | 3.883 | 210.051 | 1.000 | -12.716 | 13.828 |
| | other_pos | 1.475 | 4.575 | 249.861 | 1.000 | -14.126 | 17.076 |
| | PD_neg | -9.119 | 3.998 | 208.127 | 1.000 | -22.788 | 4.549 |
| | PD_part pos | 2.232 | 4.693 | 249.391 | 1.000 | -13.772 | 18.235 |
| | PD_pos | 7.198 | 4.038 | 252.386 | 1.000 | -6.570 | 20.966 |
| Low4_part pos | ASNB_neg | -13.263 | 5.254 | 226.078 | .810 | -31.203 | 4.676 |
| | ASNB_part pos | -4.614 | 5.874 | 247.844 | 1.000 | -24.645 | 15.418 |
| | ASNB_pos | -3.407 | 6.042 | 251.731 | 1.000 | -24.008 | 17.195 |

| | | | | | | | |
|---|---|---|---|---|---|---|---|
| | Low4_neg | -10.113 | 5.589 | 235.822 | 1.000 | -29.186 | 8.960 |
| | Low4_pos | 12.393 | 5.854 | 250.726 | 1.000 | -7.569 | 32.355 |
| | other_neg | -12.550 | 4.706 | 227.205 | .542 | -28.618 | 3.518 |
| | other_part pos | -9.557 | 4.961 | 242.106 | 1.000 | -26.479 | 7.365 |
| | other_pos | -8.638 | 5.473 | 251.717 | 1.000 | -27.298 | 10.023 |
| | PD_neg | -19.232* | 5.078 | 243.999 | **.013** | -36.551 | -1.913 |
| | PD_part pos | -7.881 | 5.435 | 227.927 | 1.000 | -26.436 | 10.674 |
| | PD_pos | -2.914 | 5.007 | 250.745 | 1.000 | -19.986 | 14.157 |
| Low4_pos | ASNB_neg | -25.656* | 4.692 | 249.994 | **<0.001** | -41.656 | -9.656 |
| | ASNB_part pos | -17.007 | 5.313 | 249.444 | .102 | -35.124 | 1.111 |
| | ASNB_pos | -15.800 | 5.403 | 250.319 | .249 | -34.224 | 2.624 |
| | Low4_neg | -22.506* | 5.027 | 249.013 | **.001** | -39.649 | -5.362 |
| | Low4_part pos | -12.393 | 5.854 | 250.726 | 1.000 | -32.355 | 7.569 |
| | other_neg | -24.943* | 4.166 | 247.793 | **<0.001** | -39.151 | -10.736 |
| | other_part pos | -21.950* | 4.334 | 252.750 | **<0.001** | -36.726 | -7.173 |
| | other_pos | -21.031* | 4.714 | 240.566 | **.001** | -37.114 | -4.947 |
| | PD_neg | -31.625* | 4.334 | 242.851 | **<0.001** | -46.409 | -16.841 |
| | PD_part pos | -20.274* | 4.963 | 253.000 | **.004** | -37.196 | -3.352 |
| | PD_pos | -15.307* | 4.361 | 249.725 | **.035** | -30.177 | -.438 |
| other_neg | ASNB_neg | -.713 | 3.225 | 218.400 | 1.000 | -11.731 | 10.305 |
| | ASNB_part pos | 7.937 | 4.213 | 252.249 | 1.000 | -6.427 | 22.301 |
| | ASNB_pos | 9.143 | 4.432 | 244.745 | 1.000 | -5.972 | 24.259 |
| | Low4_neg | 2.438 | 3.676 | 223.936 | 1.000 | -10.115 | 14.990 |
| | Low4_part pos | 12.550 | 4.706 | 227.205 | .542 | -3.518 | 28.618 |
| | Low4_pos | 24.943* | 4.166 | 247.793 | **<0.001** | 10.736 | 39.151 |
| | other_part pos | 2.994 | 2.763 | 251.850 | 1.000 | -6.426 | 12.413 |
| | other_pos | 3.913 | 3.654 | 228.401 | 1.000 | -8.562 | 16.387 |
| | PD_neg | -6.682 | 2.932 | 252.954 | 1.000 | -16.679 | 3.315 |
| | PD_part pos | 4.669 | 3.719 | 250.008 | 1.000 | -8.012 | 17.350 |

| | | | | | | | |
|---|---|---|---|---|---|---|---|
| | PD_pos | 9.636 | 2.914 | 188.312 | .074 | -.340 | 19.611 |
| other_part pos | ASNB_neg | -3.707 | 3.580 | 244.411 | 1.000 | -15.918 | 8.504 |
| | ASNB_part pos | 4.943 | 4.446 | 251.780 | 1.000 | -10.215 | 20.101 |
| | ASNB_pos | 6.150 | 4.575 | 252.576 | 1.000 | -9.448 | 21.747 |
| | Low4_neg | -.556 | 3.883 | 210.051 | 1.000 | -13.828 | 12.716 |
| | Low4_part pos | 9.557 | 4.961 | 242.106 | 1.000 | -7.365 | 26.479 |
| | Low4_pos | 21.950* | 4.334 | 252.750 | **<0.001** | 7.173 | 36.726 |
| | other_neg | -2.994 | 2.763 | 251.850 | 1.000 | -12.413 | 6.426 |
| | other_pos | .919 | 3.869 | 243.292 | 1.000 | -12.279 | 14.117 |
| | PD_neg | -9.675 | 3.231 | 252.730 | .199 | -20.690 | 1.339 |
| | PD_part pos | 1.676 | 3.917 | 252.999 | 1.000 | -11.680 | 15.031 |
| | PD_pos | 6.642 | 3.133 | 239.380 | 1.000 | -4.046 | 17.331 |
| other_pos | ASNB_neg | -4.626 | 4.288 | 252.528 | 1.000 | -19.246 | 9.994 |
| | ASNB_part pos | 4.024 | 4.973 | 251.727 | 1.000 | -12.931 | 20.978 |
| | ASNB_pos | 5.231 | 5.140 | 248.565 | 1.000 | -12.297 | 22.759 |
| | Low4_neg | -1.475 | 4.575 | 249.861 | 1.000 | -17.076 | 14.126 |
| | Low4_part pos | 8.638 | 5.473 | 251.717 | 1.000 | -10.023 | 27.298 |
| | Low4_pos | 21.031* | 4.714 | 240.566 | **.001** | 4.947 | 37.114 |
| | other_neg | -3.913 | 3.654 | 228.401 | 1.000 | -16.387 | 8.562 |
| | other_part pos | -.919 | 3.869 | 243.292 | 1.000 | -14.117 | 12.279 |
| | PD_neg | -10.594 | 3.929 | 252.777 | .493 | -23.989 | 2.800 |
| | PD_part pos | .757 | 4.556 | 249.682 | 1.000 | -14.778 | 16.291 |
| | PD_pos | 5.723 | 3.852 | 242.024 | 1.000 | -7.418 | 18.865 |
| PD_neg | ASNB_neg | 5.969 | 3.629 | 212.034 | 1.000 | -6.435 | 18.372 |
| | ASNB_part pos | 14.618 | 4.472 | 246.252 | .081 | -.634 | 29.871 |
| | ASNB_pos | 15.825 | 4.661 | 252.795 | .052 | -.064 | 31.715 |
| | Low4_neg | 9.119 | 3.998 | 208.127 | 1.000 | -4.549 | 22.788 |
| | Low4_part pos | 19.232* | 5.078 | 243.999 | **.013** | 1.913 | 36.551 |
| | Low4_pos | 31.625* | 4.334 | 242.851 | **<0.001** | 16.841 | 46.409 |

| | | | | | | | |
|---|---|---|---|---|---|---|---|
| | other_neg | 6.682 | 2.932 | 252.954 | 1.000 | -3.315 | 16.679 |
| | other_part pos | 9.675 | 3.231 | 252.730 | .199 | -1.339 | 20.690 |
| | other_pos | 10.594 | 3.929 | 252.777 | .493 | -2.800 | 23.989 |
| | PD_part pos | 11.351 | 4.076 | 252.650 | .380 | -2.546 | 25.248 |
| | PD_pos | 16.318* | 3.311 | 238.638 | **<0.001** | 5.020 | 27.615 |
| PD_part pos | ASNB_neg | -5.382 | 4.343 | 250.253 | 1.000 | -20.192 | 9.427 |
| | ASNB_part pos | 3.267 | 5.077 | 252.016 | 1.000 | -14.042 | 20.577 |
| | ASNB_pos | 4.474 | 5.128 | 249.458 | 1.000 | -13.013 | 21.961 |
| | Low4_neg | -2.232 | 4.693 | 249.391 | 1.000 | -18.235 | 13.772 |
| | Low4_part pos | 7.881 | 5.435 | 227.927 | 1.000 | -10.674 | 26.436 |
| | Low4_pos | 20.274* | 4.963 | 253.000 | **.004** | 3.352 | 37.196 |
| | other_neg | -4.669 | 3.719 | 250.008 | 1.000 | -17.350 | 8.012 |
| | other_part pos | -1.676 | 3.917 | 252.999 | 1.000 | -15.031 | 11.680 |
| | other_pos | -.757 | 4.556 | 249.682 | 1.000 | -16.291 | 14.778 |
| | PD_neg | -11.351 | 4.076 | 252.650 | .380 | -25.248 | 2.546 |
| | PD_pos | 4.967 | 3.890 | 252.278 | 1.000 | -8.295 | 18.228 |
| PD_pos | ASNB_neg | -10.349 | 3.686 | 248.901 | .355 | -22.917 | 2.220 |
| | ASNB_part pos | -1.699 | 4.475 | 247.800 | 1.000 | -16.961 | 13.563 |
| | ASNB_pos | -.492 | 4.578 | 252.837 | 1.000 | -16.099 | 15.115 |
| | Low4_neg | -7.198 | 4.038 | 252.386 | 1.000 | -20.966 | 6.570 |
| | Low4_part pos | 2.914 | 5.007 | 250.745 | 1.000 | -14.157 | 19.986 |
| | Low4_pos | 15.307* | 4.361 | 249.725 | **.035** | .438 | 30.177 |
| | other_neg | -9.636 | 2.914 | 188.312 | .074 | -19.611 | .340 |
| | other_part pos | -6.642 | 3.133 | 239.380 | 1.000 | -17.331 | 4.046 |
| | other_pos | -5.723 | 3.852 | 242.024 | 1.000 | -18.865 | 7.418 |
| | PD_neg | -16.318* | 3.311 | 238.638 | **<0.001** | -27.615 | -5.020 |
| | PD_part pos | -4.967 | 3.890 | 252.278 | 1.000 | -18.228 | 8.295 |

Based on estimated marginal means

*. The mean difference is significant at the .05 level.

a. Dependent Variable: DHDmin.

c. Adjustment for multiple comparisons: Bonferroni.

**Table S2:** Pairwise comparisons for two-way interaction model between forelimb diagnostic anaesthesia 'efficacy' and 'type' for outcome variable DHDmax, i.e. head movement asymmetry associated with changes in pushoff between contralateral forelimbs.
Given are mean differences (in mm), standard error (in mm), degrees of freedom, significance achieved, upper and lower bounds of 95% confidence intervals for the pairwise difference (in mm). Pairwise significant differences at P<0.05 after Bonferroni correction are indicated with '*'.
Forelimb block types: ASNB: abaxial sesamoid nerve block; Low4: Low4-point nerve block; PD: palmar digital nerve block; other: all other nerve/joint blocks.
Efficacy: neg: negative block (0 to 30% perceived change); part pos: partially positive block (>30 to 70% perceived change; pos: positive block (>70% change).

**Pairwise Comparisons[a]**

| (I) Type x Efficacy | (J) Type x Efficacy | Mean Difference (I-J) | Std. Error | df | Sig.[c] | 95% Confidence Interval for Difference[c] | |
|---|---|---|---|---|---|---|---|
| | | | | | | Lower Bound | Upper Bound |
| ASNB_neg | ASNB_part pos | 2.298 | 3.390 | 249.107 | 1.000 | -9.262 | 13.857 |
| | ASNB_pos | 6.951 | 3.491 | 250.760 | 1.000 | -4.953 | 18.855 |
| | Low4_neg | -.676 | 3.122 | 244.193 | 1.000 | -11.323 | 9.972 |
| | Low4_part pos | -2.786 | 3.761 | 252.989 | 1.000 | -15.607 | 10.035 |
| | Low4_pos | 12.807* | 3.303 | 247.593 | .009 | 1.544 | 24.070 |
| | other_neg | -.170 | 2.328 | 248.509 | 1.000 | -8.108 | 7.768 |
| | other_part pos | 1.882 | 2.542 | 252.827 | 1.000 | -6.785 | 10.550 |
| | other_pos | .824 | 2.993 | 244.173 | 1.000 | -9.384 | 11.031 |
| | PD_neg | -2.731 | 2.633 | 242.959 | 1.000 | -11.713 | 6.250 |
| | PD_part pos | 7.534 | 3.078 | 252.995 | .994 | -2.961 | 18.030 |
| | PD_pos | 7.583 | 2.566 | 245.059 | .227 | -1.170 | 16.337 |
| ASNB_part pos | ASNB_neg | -2.298 | 3.390 | 249.107 | 1.000 | -13.857 | 9.262 |
| | ASNB_pos | 4.653 | 3.910 | 247.854 | 1.000 | -8.680 | 17.986 |
| | Low4_neg | -2.973 | 3.601 | 251.463 | 1.000 | -15.250 | 9.303 |
| | Low4_part pos | -5.084 | 4.152 | 251.296 | 1.000 | -19.242 | 9.074 |
| | Low4_pos | 10.510 | 3.740 | 247.091 | .353 | -2.244 | 23.263 |
| | other_neg | -2.468 | 2.936 | 241.675 | 1.000 | -12.482 | 7.547 |
| | other_part pos | -.415 | 3.099 | 243.796 | 1.000 | -10.985 | 10.154 |
| | other_pos | -1.474 | 3.467 | 244.890 | 1.000 | -13.298 | 10.350 |

| | | | | | | | |
|---|---|---|---|---|---|---|---|
| | PD_neg | -5.029 | 3.174 | 252.876 | 1.000 | -15.850 | 5.791 |
| | PD_part pos | 5.237 | 3.551 | 248.650 | 1.000 | -6.873 | 17.346 |
| | PD_pos | 5.285 | 3.110 | 243.123 | 1.000 | -5.321 | 15.892 |
| ASNB_pos | ASNB_neg | -6.951 | 3.491 | 250.760 | 1.000 | -18.855 | 4.953 |
| | ASNB_part pos | -4.653 | 3.910 | 247.854 | 1.000 | -17.986 | 8.680 |
| | Low4_neg | -7.627 | 3.692 | 252.912 | 1.000 | -20.213 | 4.959 |
| | Low4_part pos | -9.737 | 4.247 | 247.901 | 1.000 | -24.221 | 4.747 |
| | Low4_pos | 5.856 | 3.821 | 252.494 | 1.000 | -7.171 | 18.883 |
| | other_neg | -7.121 | 3.063 | 234.615 | 1.000 | -17.574 | 3.332 |
| | other_part pos | -5.069 | 3.206 | 249.853 | 1.000 | -15.999 | 5.862 |
| | other_pos | -6.127 | 3.572 | 243.201 | 1.000 | -18.312 | 6.058 |
| | PD_neg | -9.682 | 3.286 | 252.549 | .232 | -20.886 | 1.522 |
| | PD_part pos | .583 | 3.637 | 252.998 | 1.000 | -11.817 | 12.984 |
| | PD_pos | .632 | 3.214 | 251.000 | 1.000 | -10.326 | 11.590 |
| Low4_neg | ASNB_neg | .676 | 3.122 | 244.193 | 1.000 | -9.972 | 11.323 |
| | ASNB_part pos | 2.973 | 3.601 | 251.463 | 1.000 | -9.303 | 15.250 |
| | ASNB_pos | 7.627 | 3.692 | 252.912 | 1.000 | -4.959 | 20.213 |
| | Low4_part pos | -2.110 | 3.968 | 249.890 | 1.000 | -15.641 | 11.420 |
| | Low4_pos | 13.483* | 3.532 | 242.600 | .011 | 1.436 | 25.530 |
| | other_neg | .506 | 2.636 | 252.733 | 1.000 | -8.481 | 9.492 |
| | other_part pos | 2.558 | 2.809 | 248.791 | 1.000 | -7.020 | 12.136 |
| | other_pos | 1.499 | 3.227 | 250.417 | 1.000 | -9.505 | 12.504 |
| | PD_neg | -2.056 | 2.902 | 244.406 | 1.000 | -11.953 | 7.841 |
| | PD_part pos | 8.210 | 3.319 | 251.995 | .927 | -3.107 | 19.527 |
| | PD_pos | 8.259 | 2.841 | 249.644 | .263 | -1.429 | 17.947 |
| Low4_part pos | ASNB_neg | 2.786 | 3.761 | 252.989 | 1.000 | -10.035 | 15.607 |
| | ASNB_part pos | 5.084 | 4.152 | 251.296 | 1.000 | -9.074 | 19.242 |
| | ASNB_pos | 9.737 | 4.247 | 247.901 | 1.000 | -4.747 | 24.221 |
| | Low4_neg | 2.110 | 3.968 | 249.890 | 1.000 | -11.420 | 15.641 |

| | | | | | | | |
|---|---|---|---|---|---|---|---|
| | Low4_pos | 15.593* | 4.101 | 239.460 | .012 | 1.600 | 29.586 |
| | other_neg | 2.616 | 3.359 | 251.993 | 1.000 | -8.837 | 14.068 |
| | other_part pos | 4.668 | 3.510 | 248.772 | 1.000 | -7.301 | 16.637 |
| | other_pos | 3.609 | 3.842 | 246.192 | 1.000 | -9.492 | 16.711 |
| | PD_neg | .054 | 3.590 | 248.875 | 1.000 | -12.188 | 12.296 |
| | PD_part pos | 10.320 | 3.898 | 252.373 | .569 | -2.970 | 23.610 |
| | PD_pos | 10.369 | 3.521 | 246.777 | .234 | -1.640 | 22.378 |
| Low4_pos | ASNB_neg | -12.807* | 3.303 | 247.593 | .009 | -24.070 | -1.544 |
| | ASNB_part pos | -10.510 | 3.740 | 247.091 | .353 | -23.263 | 2.244 |
| | ASNB_pos | -5.856 | 3.821 | 252.494 | 1.000 | -18.883 | 7.171 |
| | Low4_neg | -13.483* | 3.532 | 242.600 | .011 | -25.530 | -1.436 |
| | Low4_part pos | -15.593* | 4.101 | 239.460 | .012 | -29.586 | -1.600 |
| | other_neg | -12.977* | 2.857 | 204.454 | .001 | -22.746 | -3.209 |
| | other_part pos | -10.925* | 3.011 | 231.836 | .023 | -21.203 | -.647 |
| | other_pos | -11.984* | 3.359 | 252.956 | .028 | -23.435 | -.532 |
| | PD_neg | -15.539* | 3.079 | 252.727 | .000 | -26.035 | -5.042 |
| | PD_part pos | -5.273 | 3.472 | 244.519 | 1.000 | -17.117 | 6.571 |
| | PD_pos | -5.224 | 3.021 | 232.656 | 1.000 | -15.534 | 5.086 |
| other_neg | ASNB_neg | .170 | 2.328 | 248.509 | 1.000 | -7.768 | 8.108 |
| | ASNB_part pos | 2.468 | 2.936 | 241.675 | 1.000 | -7.547 | 12.482 |
| | ASNB_pos | 7.121 | 3.063 | 234.615 | 1.000 | -3.332 | 17.574 |
| | Low4_neg | -.506 | 2.636 | 252.733 | 1.000 | -9.492 | 8.481 |
| | Low4_part pos | -2.616 | 3.359 | 251.993 | 1.000 | -14.068 | 8.837 |
| | Low4_pos | 12.977* | 2.857 | 204.454 | .001 | 3.209 | 22.746 |
| | other_part pos | 2.052 | 1.909 | 219.995 | 1.000 | -4.467 | 8.572 |
| | other_pos | .994 | 2.481 | 198.226 | 1.000 | -7.493 | 9.480 |
| | PD_neg | -2.561 | 2.047 | 238.142 | 1.000 | -9.544 | 4.421 |
| | PD_part pos | 7.704 | 2.584 | 239.107 | .209 | -1.113 | 16.521 |
| | PD_pos | 7.753* | 1.944 | 170.904 | .007 | 1.084 | 14.422 |

| | | | | | | | |
|---|---|---|---|---|---|---|---|
| other_part pos | ASNB_neg | -1.882 | 2.542 | 252.827 | 1.000 | -10.550 | 6.785 |
| | ASNB_part pos | .415 | 3.099 | 243.796 | 1.000 | -10.154 | 10.985 |
| | ASNB_pos | 5.069 | 3.206 | 249.853 | 1.000 | -5.862 | 15.999 |
| | Low4_neg | -2.558 | 2.809 | 248.791 | 1.000 | -12.136 | 7.020 |
| | Low4_part pos | -4.668 | 3.510 | 248.772 | 1.000 | -16.637 | 7.301 |
| | Low4_pos | 10.925* | 3.011 | 231.836 | .023 | .647 | 21.203 |
| | other_neg | -2.052 | 1.909 | 219.995 | 1.000 | -8.572 | 4.467 |
| | other_pos | -1.059 | 2.663 | 226.068 | 1.000 | -10.151 | 8.033 |
| | PD_neg | -4.614 | 2.268 | 248.973 | 1.000 | -12.349 | 3.121 |
| | PD_part pos | 5.652 | 2.756 | 251.766 | 1.000 | -3.743 | 15.047 |
| | PD_pos | 5.701 | 2.163 | 236.754 | .591 | -1.680 | 13.081 |
| other_pos | ASNB_neg | -.824 | 2.993 | 244.173 | 1.000 | -11.031 | 9.384 |
| | ASNB_part pos | 1.474 | 3.467 | 244.890 | 1.000 | -10.350 | 13.298 |
| | ASNB_pos | 6.127 | 3.572 | 243.201 | 1.000 | -6.058 | 18.312 |
| | Low4_neg | -1.499 | 3.227 | 250.417 | 1.000 | -12.504 | 9.505 |
| | Low4_part pos | -3.609 | 3.842 | 246.192 | 1.000 | -16.711 | 9.492 |
| | Low4_pos | 11.984* | 3.359 | 252.956 | .028 | .532 | 23.435 |
| | other_neg | -.994 | 2.481 | 198.226 | 1.000 | -9.480 | 7.493 |
| | other_part pos | 1.059 | 2.663 | 226.068 | 1.000 | -8.033 | 10.151 |
| | PD_neg | -3.555 | 2.750 | 248.663 | 1.000 | -12.934 | 5.824 |
| | PD_part pos | 6.711 | 3.173 | 245.532 | 1.000 | -4.110 | 17.532 |
| | PD_pos | 6.760 | 2.665 | 240.066 | .780 | -2.332 | 15.851 |
| PD_neg | ASNB_neg | 2.731 | 2.633 | 242.959 | 1.000 | -6.250 | 11.713 |
| | ASNB_part pos | 5.029 | 3.174 | 252.876 | 1.000 | -5.791 | 15.850 |
| | ASNB_pos | 9.682 | 3.286 | 252.549 | .232 | -1.522 | 20.886 |
| | Low4_neg | 2.056 | 2.902 | 244.406 | 1.000 | -7.841 | 11.953 |
| | Low4_part pos | -.054 | 3.590 | 248.875 | 1.000 | -12.296 | 12.188 |
| | Low4_pos | 15.539* | 3.079 | 252.727 | .000 | 5.042 | 26.035 |
| | other_neg | 2.561 | 2.047 | 238.142 | 1.000 | -4.421 | 9.544 |

| | | | | | | | |
|---|---|---|---|---|---|---|---|
| | other_part pos | 4.614 | 2.268 | 248.973 | 1.000 | -3.121 | 12.349 |
| | other_pos | 3.555 | 2.750 | 248.663 | 1.000 | -5.824 | 12.934 |
| | PD_part pos | 10.266* | 2.859 | 250.598 | .026 | .516 | 20.016 |
| | PD_pos | 10.315* | 2.289 | 240.489 | .001 | 2.504 | 18.125 |
| PD_part pos | ASNB_neg | -7.534 | 3.078 | 252.995 | .994 | -18.030 | 2.961 |
| | ASNB_part pos | -5.237 | 3.551 | 248.650 | 1.000 | -17.346 | 6.873 |
| | ASNB_pos | -.583 | 3.637 | 252.998 | 1.000 | -12.984 | 11.817 |
| | Low4_neg | -8.210 | 3.319 | 251.995 | .927 | -19.527 | 3.107 |
| | Low4_part pos | -10.320 | 3.898 | 252.373 | .569 | -23.610 | 2.970 |
| | Low4_pos | 5.273 | 3.472 | 244.519 | 1.000 | -6.571 | 17.117 |
| | other_neg | -7.704 | 2.584 | 239.107 | .209 | -16.521 | 1.113 |
| | other_part pos | -5.652 | 2.756 | 251.766 | 1.000 | -15.047 | 3.743 |
| | other_pos | -6.711 | 3.173 | 245.532 | 1.000 | -17.532 | 4.110 |
| | PD_neg | -10.266* | 2.859 | 250.598 | .026 | -20.016 | -.516 |
| | PD_pos | .049 | 2.756 | 252.494 | 1.000 | -9.349 | 9.447 |
| PD_pos | ASNB_neg | -7.583 | 2.566 | 245.059 | .227 | -16.337 | 1.170 |
| | ASNB_part pos | -5.285 | 3.110 | 243.123 | 1.000 | -15.892 | 5.321 |
| | ASNB_pos | -.632 | 3.214 | 251.000 | 1.000 | -11.590 | 10.326 |
| | Low4_neg | -8.259 | 2.841 | 249.644 | .263 | -17.947 | 1.429 |
| | Low4_part pos | -10.369 | 3.521 | 246.777 | .234 | -22.378 | 1.640 |
| | Low4_pos | 5.224 | 3.021 | 232.656 | 1.000 | -5.086 | 15.534 |
| | other_neg | -7.753* | 1.944 | 170.904 | .007 | -14.422 | -1.084 |
| | other_part pos | -5.701 | 2.163 | 236.754 | .591 | -13.081 | 1.680 |
| | other_pos | -6.760 | 2.665 | 240.066 | .780 | -15.851 | 2.332 |
| | PD_neg | -10.315* | 2.289 | 240.489 | .001 | -18.125 | -2.504 |
| | PD_part pos | -.049 | 2.756 | 252.494 | 1.000 | -9.447 | 9.349 |

Based on estimated marginal means

*. The mean difference is significant at the .05 level.

a. Dependent Variable: DHDmax.

c. Adjustment for multiple comparisons: Bonferroni.

**Table S3:** Pairwise comparisons for two-way interaction model between forelimb diagnostic anaesthesia ‘efficacy’ and ‘type’ for outcome variable DHDup, i.e. head movement asymmetry associated with changes in upward movement amplitudes between stride halves.
Given are mean differences (in mm), standard error (in mm), degrees of freedom, significance achieved, upper and lower bounds of 95% confidence intervals for the pairwise difference (in mm).
Pairwise significant differences at P<0.05 after Bonferroni correction are indicated with ‘*’.
Forelimb block types: ASNB: abaxial sesamoid nerve block; Low4: Low4-point nerve block; PD: palmar digital nerve block; other: all other nerve/joint blocks.
Efficacy: neg: negative block (0 to 30% perceived change); part pos: partially positive block (>30 to 70% perceived change; pos: positive block (>70% change).

**Pairwise Comparisons[a]**

| (I) Type x Efficacy | (J) Type x Efficacy | Mean Difference (I-J) | Std. Error | df | Sig.[c] | 95% Confidence Interval for Difference[c] Lower Bound | Upper Bound |
|---|---|---|---|---|---|---|---|
| ASNB_neg | ASNB_part pos | 10.281 | 7.347 | 252.084 | 1.000 | -14.771 | 35.332 |
| | ASNB_pos | 17.230 | 7.562 | 252.090 | 1.000 | -8.552 | 43.012 |
| | Low4_neg | 2.490 | 6.670 | 231.882 | 1.000 | -20.277 | 25.256 |
| | Low4_part pos | 9.162 | 8.096 | 247.992 | 1.000 | -18.447 | 36.771 |
| | Low4_pos | 36.713* | 7.160 | 252.492 | .000 | 12.302 | 61.125 |
| | other_neg | .560 | 4.987 | 239.662 | 1.000 | -16.453 | 17.574 |
| | other_part pos | 5.566 | 5.485 | 252.212 | 1.000 | -13.136 | 24.268 |
| | other_pos | 4.077 | 6.506 | 248.277 | 1.000 | -18.110 | 26.264 |
| | PD_neg | -7.460 | 5.625 | 232.598 | 1.000 | -26.658 | 11.738 |
| | PD_part pos | 12.611 | 6.641 | 252.558 | 1.000 | -10.030 | 35.252 |
| | PD_pos | 16.760 | 5.580 | 247.080 | .194 | -2.271 | 35.790 |
| ASNB_part pos | ASNB_neg | -10.281 | 7.347 | 252.084 | 1.000 | -35.332 | 14.771 |
| | ASNB_pos | 6.949 | 8.489 | 249.310 | 1.000 | -21.997 | 35.895 |
| | Low4_neg | -7.791 | 7.785 | 252.973 | 1.000 | -34.334 | 18.751 |
| | Low4_part pos | -1.118 | 8.980 | 252.996 | 1.000 | -31.736 | 29.499 |
| | Low4_pos | 26.433 | 8.109 | 252.477 | .084 | -1.214 | 54.079 |
| | other_neg | -9.720 | 6.388 | 247.129 | 1.000 | -31.506 | 12.066 |
| | other_part pos | -4.715 | 6.739 | 247.321 | 1.000 | -27.698 | 18.269 |

| | | | | | | | |
|---|---|---|---|---|---|---|---|
| | other_pos | -6.204 | 7.538 | 247.429 | 1.000 | -31.909 | 19.502 |
| | PD_neg | -17.741 | 6.848 | 252.355 | .669 | -41.089 | 5.608 |
| | PD_part pos | 2.331 | 7.705 | 250.081 | 1.000 | -23.944 | 28.605 |
| | PD_pos | 6.479 | 6.768 | 244.886 | 1.000 | -16.606 | 29.563 |
| ASNB_pos | ASNB_neg | -17.230 | 7.562 | 252.090 | 1.000 | -43.012 | 8.552 |
| | ASNB_part pos | -6.949 | 8.489 | 249.310 | 1.000 | -35.895 | 21.997 |
| | Low4_neg | -14.740 | 7.968 | 252.747 | 1.000 | -41.906 | 12.426 |
| | Low4_part pos | -8.068 | 9.209 | 252.159 | 1.000 | -39.467 | 23.331 |
| | Low4_pos | 19.484 | 8.255 | 252.964 | 1.000 | -8.661 | 47.628 |
| | other_neg | -16.669 | 6.687 | 239.029 | .881 | -39.483 | 6.144 |
| | other_part pos | -11.664 | 6.950 | 251.074 | 1.000 | -35.362 | 12.034 |
| | other_pos | -13.153 | 7.775 | 244.940 | 1.000 | -39.672 | 13.366 |
| | PD_neg | -24.690* | 7.104 | 252.927 | .040 | -48.909 | -.471 |
| | PD_part pos | -4.618 | 7.844 | 252.440 | 1.000 | -31.364 | 22.127 |
| | PD_pos | -.470 | 6.961 | 251.928 | 1.000 | -24.204 | 23.263 |
| Low4_neg | ASNB_neg | -2.490 | 6.670 | 231.882 | 1.000 | -25.256 | 20.277 |
| | ASNB_part pos | 7.791 | 7.785 | 252.973 | 1.000 | -18.751 | 34.334 |
| | ASNB_pos | 14.740 | 7.968 | 252.747 | 1.000 | -12.426 | 41.906 |
| | Low4_part pos | 6.673 | 8.579 | 252.399 | 1.000 | -22.576 | 35.922 |
| | Low4_pos | 34.224* | 7.670 | 251.830 | .001 | 8.073 | 60.375 |
| | other_neg | -1.929 | 5.668 | 246.612 | 1.000 | -21.260 | 17.402 |
| | other_part pos | 3.076 | 6.014 | 236.927 | 1.000 | -17.446 | 23.599 |
| | other_pos | 1.588 | 6.986 | 252.904 | 1.000 | -22.230 | 25.405 |
| | PD_neg | -9.950 | 6.201 | 232.278 | 1.000 | -31.113 | 11.214 |
| | PD_part pos | 10.122 | 7.173 | 252.951 | 1.000 | -14.335 | 34.579 |
| | PD_pos | 14.270 | 6.154 | 252.535 | 1.000 | -6.713 | 35.253 |
| Low4_part pos | ASNB_neg | -9.162 | 8.096 | 247.992 | 1.000 | -36.771 | 18.447 |
| | ASNB_part pos | 1.118 | 8.980 | 252.996 | 1.000 | -29.499 | 31.736 |
| | ASNB_pos | 8.068 | 9.209 | 252.159 | 1.000 | -23.331 | 39.467 |

| | | | | | | | |
|---|---|---|---|---|---|---|---|
| | Low4_neg | -6.673 | 8.579 | 252.399 | 1.000 | -35.922 | 22.576 |
| | Low4_pos | 27.551 | 8.919 | 250.487 | .147 | -2.860 | 57.963 |
| | other_neg | -8.602 | 7.245 | 249.992 | 1.000 | -33.307 | 16.103 |
| | other_part pos | -3.596 | 7.597 | 252.965 | 1.000 | -29.498 | 22.305 |
| | other_pos | -5.085 | 8.337 | 251.522 | 1.000 | -33.512 | 23.342 |
| | PD_neg | -16.622 | 7.771 | 252.995 | 1.000 | -43.118 | 9.873 |
| | PD_part pos | 3.449 | 8.377 | 246.113 | 1.000 | -25.121 | 32.020 |
| | PD_pos | 7.597 | 7.636 | 252.345 | 1.000 | -18.439 | 33.634 |
| Low4_pos | ASNB_neg | -36.713* | 7.160 | 252.492 | .000 | -61.125 | -12.302 |
| | ASNB_part pos | -26.433 | 8.109 | 252.477 | .084 | -54.079 | 1.214 |
| | ASNB_pos | -19.484 | 8.255 | 252.964 | 1.000 | -47.628 | 8.661 |
| | Low4_neg | -34.224* | 7.670 | 251.830 | .001 | -60.375 | -8.073 |
| | Low4_part pos | -27.551 | 8.919 | 250.487 | .147 | -57.963 | 2.860 |
| | other_neg | -36.153* | 6.277 | 226.800 | .000 | -57.584 | -14.722 |
| | other_part pos | -31.148* | 6.570 | 244.145 | .000 | -53.557 | -8.738 |
| | other_pos | -32.636* | 7.236 | 250.857 | .001 | -57.309 | -7.963 |
| | PD_neg | -44.174* | 6.644 | 252.142 | .000 | -66.826 | -21.521 |
| | PD_part pos | -24.102 | 7.544 | 250.026 | .104 | -49.826 | 1.622 |
| | PD_pos | -19.954 | 6.593 | 241.321 | .181 | -42.447 | 2.539 |
| other_neg | ASNB_neg | -.560 | 4.987 | 239.662 | 1.000 | -17.574 | 16.453 |
| | ASNB_part pos | 9.720 | 6.388 | 247.129 | 1.000 | -12.066 | 31.506 |
| | ASNB_pos | 16.669 | 6.687 | 239.029 | .881 | -6.144 | 39.483 |
| | Low4_neg | 1.929 | 5.668 | 246.612 | 1.000 | -17.402 | 21.260 |
| | Low4_part pos | 8.602 | 7.245 | 249.992 | 1.000 | -16.103 | 33.307 |
| | Low4_pos | 36.153* | 6.277 | 226.800 | .000 | 14.722 | 57.584 |
| | other_part pos | 5.006 | 4.178 | 237.500 | 1.000 | -9.249 | 19.260 |
| | other_pos | 3.517 | 5.468 | 210.501 | 1.000 | -15.172 | 22.206 |
| | PD_neg | -8.020 | 4.455 | 248.281 | 1.000 | -23.212 | 7.171 |
| | PD_part pos | 12.051 | 5.629 | 245.054 | 1.000 | -7.148 | 31.250 |

| | | | | | | | |
|---|---|---|---|---|---|---|---|
| | PD_pos | 16.199* | 4.317 | 180.858 | .016 | 1.409 | 30.990 |
| other_part pos | ASNB_neg | -5.566 | 5.485 | 252.212 | 1.000 | -24.268 | 13.136 |
| | ASNB_part pos | 4.715 | 6.739 | 247.321 | 1.000 | -18.269 | 27.698 |
| | ASNB_pos | 11.664 | 6.950 | 251.074 | 1.000 | -12.034 | 35.362 |
| | Low4_neg | -3.076 | 6.014 | 236.927 | 1.000 | -23.599 | 17.446 |
| | Low4_part pos | 3.596 | 7.597 | 252.965 | 1.000 | -22.305 | 29.498 |
| | Low4_pos | 31.148* | 6.570 | 244.145 | .000 | 8.738 | 53.557 |
| | other_neg | -5.006 | 4.178 | 237.500 | 1.000 | -19.260 | 9.249 |
| | other_pos | -1.489 | 5.828 | 232.705 | 1.000 | -21.379 | 18.402 |
| | PD_neg | -13.026 | 4.918 | 251.794 | .567 | -29.794 | 3.742 |
| | PD_part pos | 7.045 | 5.963 | 252.524 | 1.000 | -13.286 | 27.377 |
| | PD_pos | 11.194 | 4.720 | 237.998 | 1.000 | -4.912 | 27.300 |
| other_pos | ASNB_neg | -4.077 | 6.506 | 248.277 | 1.000 | -26.264 | 18.110 |
| | ASNB_part pos | 6.204 | 7.538 | 247.429 | 1.000 | -19.502 | 31.909 |
| | ASNB_pos | 13.153 | 7.775 | 244.940 | 1.000 | -13.366 | 39.672 |
| | Low4_neg | -1.588 | 6.986 | 252.904 | 1.000 | -25.405 | 22.230 |
| | Low4_part pos | 5.085 | 8.337 | 251.522 | 1.000 | -23.342 | 33.512 |
| | Low4_pos | 32.636* | 7.236 | 250.857 | .001 | 7.963 | 57.309 |
| | other_neg | -3.517 | 5.468 | 210.501 | 1.000 | -22.206 | 15.172 |
| | other_part pos | 1.489 | 5.828 | 232.705 | 1.000 | -18.402 | 21.379 |
| | PD_neg | -11.537 | 5.968 | 250.428 | 1.000 | -31.886 | 8.811 |
| | PD_part pos | 8.534 | 6.898 | 246.937 | 1.000 | -14.991 | 32.060 |
| | PD_pos | 12.682 | 5.810 | 240.032 | 1.000 | -7.139 | 32.504 |
| PD_neg | ASNB_neg | 7.460 | 5.625 | 232.598 | 1.000 | -11.738 | 26.658 |
| | ASNB_part pos | 17.741 | 6.848 | 252.355 | .669 | -5.608 | 41.089 |
| | ASNB_pos | 24.690* | 7.104 | 252.927 | .040 | .471 | 48.909 |
| | Low4_neg | 9.950 | 6.201 | 232.278 | 1.000 | -11.214 | 31.113 |
| | Low4_part pos | 16.622 | 7.771 | 252.995 | 1.000 | -9.873 | 43.118 |
| | Low4_pos | 44.174* | 6.644 | 252.142 | .000 | 21.521 | 66.826 |

| | | | | | | | |
|---|---|---|---|---|---|---|---|
| | other_neg | 8.020 | 4.455 | 248.281 | 1.000 | -7.171 | 23.212 |
| | other_part pos | 13.026 | 4.918 | 251.794 | .567 | -3.742 | 29.794 |
| | other_pos | 11.537 | 5.968 | 250.428 | 1.000 | -8.811 | 31.886 |
| | PD_part pos | 20.071 | 6.196 | 251.680 | .090 | -1.053 | 41.196 |
| | PD_pos | 24.220* | 4.991 | 240.113 | .000 | 7.193 | 41.246 |
| PD_part pos | ASNB_neg | -12.611 | 6.641 | 252.558 | 1.000 | -35.252 | 10.030 |
| | ASNB_part pos | -2.331 | 7.705 | 250.081 | 1.000 | -28.605 | 23.944 |
| | ASNB_pos | 4.618 | 7.844 | 252.440 | 1.000 | -22.127 | 31.364 |
| | Low4_neg | -10.122 | 7.173 | 252.951 | 1.000 | -34.579 | 14.335 |
| | Low4_part pos | -3.449 | 8.377 | 246.113 | 1.000 | -32.020 | 25.121 |
| | Low4_pos | 24.102 | 7.544 | 250.026 | .104 | -1.622 | 49.826 |
| | other_neg | -12.051 | 5.629 | 245.054 | 1.000 | -31.250 | 7.148 |
| | other_part pos | -7.045 | 5.963 | 252.524 | 1.000 | -27.377 | 13.286 |
| | other_pos | -8.534 | 6.898 | 246.937 | 1.000 | -32.060 | 14.991 |
| | PD_neg | -20.071 | 6.196 | 251.680 | .090 | -41.196 | 1.053 |
| | PD_pos | 4.148 | 5.940 | 252.556 | 1.000 | -16.105 | 24.401 |
| PD_pos | ASNB_neg | -16.760 | 5.580 | 247.080 | .194 | -35.790 | 2.271 |
| | ASNB_part pos | -6.479 | 6.768 | 244.886 | 1.000 | -29.563 | 16.606 |
| | ASNB_pos | .470 | 6.961 | 251.928 | 1.000 | -23.263 | 24.204 |
| | Low4_neg | -14.270 | 6.154 | 252.535 | 1.000 | -35.253 | 6.713 |
| | Low4_part pos | -7.597 | 7.636 | 252.345 | 1.000 | -33.634 | 18.439 |
| | Low4_pos | 19.954 | 6.593 | 241.321 | .181 | -2.539 | 42.447 |
| | other_neg | -16.199* | 4.317 | 180.858 | .016 | -30.990 | -1.409 |
| | other_part pos | -11.194 | 4.720 | 237.998 | 1.000 | -27.300 | 4.912 |
| | other_pos | -12.682 | 5.810 | 240.032 | 1.000 | -32.504 | 7.139 |
| | PD_neg | -24.220* | 4.991 | 240.113 | .000 | -41.246 | -7.193 |
| | PD_part pos | -4.148 | 5.940 | 252.556 | 1.000 | -24.401 | 16.105 |

Based on estimated marginal means

*. The mean difference is significant at the .05 level.

a. Dependent Variable: DHDup.

c. Adjustment for multiple comparisons: Bonferroni.

**Table S4:** Pairwise comparisons for two-way interaction model between forelimb diagnostic anaesthesia 'efficacy' and 'type' for outcome variable DPDmax, i.e. compensatory pelvic movement asymmetry associated with changes in pushoff between contralateral hind limbs.
Given are mean differences (in mm), standard error (in mm), degrees of freedom, significance achieved, upper and lower bounds of 95% confidence intervals for the pairwise difference (in mm).
Pairwise significant differences at P<0.05 after Bonferroni correction are indicated with '*'.
Forelimb block types: ASNB: abaxial sesamoid nerve block; Low4: Low4-point nerve block; PD: palmar digital nerve block; other: all other nerve/joint blocks.
Efficacy: neg: negative block (0 to 30% perceived change); part pos: partially positive block (>30 to 70% perceived change; pos: positive block (>70% change).

**Pairwise Comparisons**[a]

| (I) Type x Efficacy | (J) Type x Efficacy | Mean Difference (I-J) | Std. Error | df | Sig.[c] | 95% Confidence Interval for Difference[c] Lower Bound | Upper Bound |
|---|---|---|---|---|---|---|---|
| ASNB_neg | ASNB_part pos | -.262 | 1.568 | 218.946 | 1.000 | -5.617 | 5.094 |
| | ASNB_pos | 2.661 | 1.647 | 235.621 | 1.000 | -2.960 | 8.282 |
| | Low4_neg | 2.198 | 1.310 | 180.401 | 1.000 | -2.289 | 6.684 |
| | Low4_part pos | 1.402 | 1.623 | 186.797 | 1.000 | -4.154 | 6.959 |
| | Low4_pos | -2.830 | 1.502 | 208.871 | 1.000 | -7.965 | 2.305 |
| | other_neg | 1.699 | .995 | 187.928 | 1.000 | -1.707 | 5.104 |
| | other_part pos | .626 | 1.137 | 205.055 | 1.000 | -3.264 | 4.515 |
| | other_pos | .535 | 1.411 | 225.271 | 1.000 | -4.281 | 5.352 |
| | PD_neg | 2.458 | 1.119 | 190.911 | 1.000 | -1.372 | 6.289 |
| | PD_part pos | .114 | 1.424 | 233.320 | 1.000 | -4.746 | 4.974 |
| | PD_pos | -1.796 | 1.256 | 249.395 | 1.000 | -6.079 | 2.487 |
| ASNB_part pos | ASNB_neg | .262 | 1.568 | 218.946 | 1.000 | -5.094 | 5.617 |
| | ASNB_pos | 2.922 | 1.869 | 237.418 | 1.000 | -3.454 | 9.299 |
| | Low4_neg | 2.459 | 1.628 | 209.074 | 1.000 | -3.104 | 8.022 |
| | Low4_part pos | 1.664 | 1.875 | 207.647 | 1.000 | -4.746 | 8.073 |
| | Low4_pos | -2.569 | 1.696 | 206.561 | 1.000 | -8.366 | 3.229 |
| | other_neg | 1.960 | 1.386 | 224.452 | 1.000 | -2.773 | 6.694 |
| | other_part pos | .887 | 1.469 | 227.022 | 1.000 | -4.127 | 5.902 |

| | | | | | | | |
|---|---|---|---|---|---|---|---|
| | other_pos | .797 | 1.643 | 227.442 | 1.000 | -4.813 | 6.407 |
| | PD_neg | 2.720 | 1.432 | 211.921 | 1.000 | -2.173 | 7.613 |
| | PD_part pos | .376 | 1.698 | 239.298 | 1.000 | -5.417 | 6.168 |
| | PD_pos | -1.534 | 1.517 | 245.267 | 1.000 | -6.707 | 3.638 |
| ASNB_pos | ASNB_neg | -2.661 | 1.647 | 235.621 | 1.000 | -8.282 | 2.960 |
| | ASNB_part pos | -2.922 | 1.869 | 237.418 | 1.000 | -9.299 | 3.454 |
| | Low4_neg | -.463 | 1.694 | 225.425 | 1.000 | -6.248 | 5.322 |
| | Low4_part pos | -1.259 | 1.956 | 218.650 | 1.000 | -7.941 | 5.424 |
| | Low4_pos | -5.491 | 1.756 | 224.581 | .132 | -11.488 | .506 |
| | other_neg | -.962 | 1.500 | 242.356 | 1.000 | -6.081 | 4.156 |
| | other_part pos | -2.035 | 1.524 | 237.225 | 1.000 | -7.237 | 3.166 |
| | other_pos | -2.126 | 1.730 | 240.179 | 1.000 | -8.027 | 3.776 |
| | PD_neg | -.203 | 1.544 | 236.966 | 1.000 | -5.471 | 5.066 |
| | PD_part pos | -2.547 | 1.672 | 228.085 | 1.000 | -8.254 | 3.160 |
| | PD_pos | -4.457 | 1.530 | 241.322 | .259 | -9.677 | .763 |
| Low4_neg | ASNB_neg | -2.198 | 1.310 | 180.401 | 1.000 | -6.684 | 2.289 |
| | ASNB_part pos | -2.459 | 1.628 | 209.074 | 1.000 | -8.022 | 3.104 |
| | ASNB_pos | .463 | 1.694 | 225.425 | 1.000 | -5.322 | 6.248 |
| | Low4_part pos | -.795 | 1.734 | 186.548 | 1.000 | -6.734 | 5.143 |
| | Low4_pos | -5.028 | 1.595 | 201.007 | .123 | -10.484 | .428 |
| | other_neg | -.499 | 1.130 | 182.301 | 1.000 | -4.370 | 3.373 |
| | other_part pos | -1.572 | 1.184 | 180.419 | 1.000 | -5.630 | 2.486 |
| | other_pos | -1.662 | 1.468 | 210.995 | 1.000 | -6.680 | 3.356 |
| | PD_neg | .261 | 1.224 | 184.858 | 1.000 | -3.931 | 4.453 |
| | PD_part pos | -2.084 | 1.522 | 224.345 | 1.000 | -7.280 | 3.113 |
| | PD_pos | -3.993 | 1.329 | 235.430 | .195 | -8.529 | .543 |
| Low4_part pos | ASNB_neg | -1.402 | 1.623 | 186.797 | 1.000 | -6.959 | 4.154 |
| | ASNB_part pos | -1.664 | 1.875 | 207.647 | 1.000 | -8.073 | 4.746 |
| | ASNB_pos | 1.259 | 1.956 | 218.650 | 1.000 | -5.424 | 7.941 |

| | | | | | | | |
|---|---|---|---|---|---|---|---|
| | Low4_neg | .795 | 1.734 | 186.548 | 1.000 | -5.143 | 6.734 |
| | Low4_pos | -4.232 | 1.864 | 202.354 | 1.000 | -10.607 | 2.142 |
| | other_neg | .296 | 1.449 | 183.479 | 1.000 | -4.668 | 5.261 |
| | other_part pos | -.777 | 1.554 | 192.613 | 1.000 | -6.096 | 4.543 |
| | other_pos | -.867 | 1.759 | 209.780 | 1.000 | -6.878 | 5.144 |
| | PD_neg | 1.056 | 1.600 | 198.158 | 1.000 | -4.419 | 6.531 |
| | PD_part pos | -1.288 | 1.697 | 198.961 | 1.000 | -7.094 | 4.517 |
| | PD_pos | -3.198 | 1.617 | 219.603 | 1.000 | -8.721 | 2.325 |
| Low4_pos | ASNB_neg | 2.830 | 1.502 | 208.871 | 1.000 | -2.305 | 7.965 |
| | ASNB_part pos | 2.569 | 1.696 | 206.561 | 1.000 | -3.229 | 8.366 |
| | ASNB_pos | 5.491 | 1.756 | 224.581 | .132 | -.506 | 11.488 |
| | Low4_neg | 5.028 | 1.595 | 201.007 | .123 | -.428 | 10.484 |
| | Low4_part pos | 4.232 | 1.864 | 202.354 | 1.000 | -2.142 | 10.607 |
| | other_neg | 4.529 | 1.367 | 217.476 | .071 | -.139 | 9.197 |
| | other_part pos | 3.456 | 1.410 | 215.723 | .992 | -1.361 | 8.272 |
| | other_pos | 3.366 | 1.492 | 204.452 | 1.000 | -1.736 | 8.467 |
| | PD_neg | 5.289* | 1.373 | 204.361 | .010 | .592 | 9.985 |
| | PD_part pos | 2.944 | 1.631 | 229.566 | 1.000 | -2.625 | 8.513 |
| | PD_pos | 1.034 | 1.457 | 238.517 | 1.000 | -3.938 | 6.007 |
| other_neg | ASNB_neg | -1.699 | .995 | 187.928 | 1.000 | -5.104 | 1.707 |
| | ASNB_part pos | -1.960 | 1.386 | 224.452 | 1.000 | -6.694 | 2.773 |
| | ASNB_pos | .962 | 1.500 | 242.356 | 1.000 | -4.156 | 6.081 |
| | Low4_neg | .499 | 1.130 | 182.301 | 1.000 | -3.373 | 4.370 |
| | Low4_part pos | -.296 | 1.449 | 183.479 | 1.000 | -5.261 | 4.668 |
| | Low4_pos | -4.529 | 1.367 | 217.476 | .071 | -9.197 | .139 |
| | other_part pos | -1.073 | .896 | 209.597 | 1.000 | -4.135 | 1.989 |
| | other_pos | -1.163 | 1.237 | 234.340 | 1.000 | -5.384 | 3.058 |
| | PD_neg | .760 | .950 | 214.047 | 1.000 | -2.486 | 4.005 |
| | PD_part pos | -1.585 | 1.250 | 241.505 | 1.000 | -5.848 | 2.679 |

| | | | | | | | |
|---|---|---|---|---|---|---|---|
| | PD_pos | -3.494 | 1.047 | 252.593 | .064 | -7.064 | .075 |
| other_part pos | ASNB_neg | -.626 | 1.137 | 205.055 | 1.000 | -4.515 | 3.264 |
| | ASNB_part pos | -.887 | 1.469 | 227.022 | 1.000 | -5.902 | 4.127 |
| | ASNB_pos | 2.035 | 1.524 | 237.225 | 1.000 | -3.166 | 7.237 |
| | Low4_neg | 1.572 | 1.184 | 180.419 | 1.000 | -2.486 | 5.630 |
| | Low4_part pos | .777 | 1.554 | 192.613 | 1.000 | -4.543 | 6.096 |
| | Low4_pos | -3.456 | 1.410 | 215.723 | .992 | -8.272 | 1.361 |
| | other_neg | 1.073 | .896 | 209.597 | 1.000 | -1.989 | 4.135 |
| | other_pos | -.090 | 1.294 | 230.811 | 1.000 | -4.507 | 4.327 |
| | PD_neg | 1.833 | 1.052 | 219.614 | 1.000 | -1.759 | 5.425 |
| | PD_part pos | -.512 | 1.301 | 237.180 | 1.000 | -4.950 | 3.927 |
| | PD_pos | -2.421 | 1.081 | 250.977 | 1.000 | -6.108 | 1.266 |
| other_pos | ASNB_neg | -.535 | 1.411 | 225.271 | 1.000 | -5.352 | 4.281 |
| | ASNB_part pos | -.797 | 1.643 | 227.442 | 1.000 | -6.407 | 4.813 |
| | ASNB_pos | 2.126 | 1.730 | 240.179 | 1.000 | -3.776 | 8.027 |
| | Low4_neg | 1.662 | 1.468 | 210.995 | 1.000 | -3.356 | 6.680 |
| | Low4_part pos | .867 | 1.759 | 209.780 | 1.000 | -5.144 | 6.878 |
| | Low4_pos | -3.366 | 1.492 | 204.452 | 1.000 | -8.467 | 1.736 |
| | other_neg | 1.163 | 1.237 | 234.340 | 1.000 | -3.058 | 5.384 |
| | other_part pos | .090 | 1.294 | 230.811 | 1.000 | -4.327 | 4.507 |
| | PD_neg | 1.923 | 1.295 | 227.929 | 1.000 | -2.499 | 6.345 |
| | PD_part pos | -.421 | 1.533 | 241.373 | 1.000 | -5.652 | 4.809 |
| | PD_pos | -2.331 | 1.318 | 247.957 | 1.000 | -6.828 | 2.165 |
| PD_neg | ASNB_neg | -2.458 | 1.119 | 190.911 | 1.000 | -6.289 | 1.372 |
| | ASNB_part pos | -2.720 | 1.432 | 211.921 | 1.000 | -7.613 | 2.173 |
| | ASNB_pos | .203 | 1.544 | 236.966 | 1.000 | -5.066 | 5.471 |
| | Low4_neg | -.261 | 1.224 | 184.858 | 1.000 | -4.453 | 3.931 |
| | Low4_part pos | -1.056 | 1.600 | 198.158 | 1.000 | -6.531 | 4.419 |
| | Low4_pos | -5.289* | 1.373 | 204.361 | .010 | -9.985 | -.592 |

| | | | | | | | |
|---|---|---|---|---|---|---|---|
| | other_neg | -.760 | .950 | 214.047 | 1.000 | -4.005 | 2.486 |
| | other_part pos | -1.833 | 1.052 | 219.614 | 1.000 | -5.425 | 1.759 |
| | other_pos | -1.923 | 1.295 | 227.929 | 1.000 | -6.345 | 2.499 |
| | PD_part pos | -2.344 | 1.367 | 243.169 | 1.000 | -7.007 | 2.319 |
| | PD_pos | -4.254* | 1.154 | 252.915 | .018 | -8.189 | -.319 |
| PD_part pos | ASNB_neg | -.114 | 1.424 | 233.320 | 1.000 | -4.974 | 4.746 |
| | ASNB_part pos | -.376 | 1.698 | 239.298 | 1.000 | -6.168 | 5.417 |
| | ASNB_pos | 2.547 | 1.672 | 228.085 | 1.000 | -3.160 | 8.254 |
| | Low4_neg | 2.084 | 1.522 | 224.345 | 1.000 | -3.113 | 7.280 |
| | Low4_part pos | 1.288 | 1.697 | 198.961 | 1.000 | -4.517 | 7.094 |
| | Low4_pos | -2.944 | 1.631 | 229.566 | 1.000 | -8.513 | 2.625 |
| | other_neg | 1.585 | 1.250 | 241.505 | 1.000 | -2.679 | 5.848 |
| | other_part pos | .512 | 1.301 | 237.180 | 1.000 | -3.927 | 4.950 |
| | other_pos | .421 | 1.533 | 241.373 | 1.000 | -4.809 | 5.652 |
| | PD_neg | 2.344 | 1.367 | 243.169 | 1.000 | -2.319 | 7.007 |
| | PD_pos | -1.910 | 1.295 | 242.444 | 1.000 | -6.328 | 2.509 |
| PD_pos | ASNB_neg | 1.796 | 1.256 | 249.395 | 1.000 | -2.487 | 6.079 |
| | ASNB_part pos | 1.534 | 1.517 | 245.267 | 1.000 | -3.638 | 6.707 |
| | ASNB_pos | 4.457 | 1.530 | 241.322 | .259 | -.763 | 9.677 |
| | Low4_neg | 3.993 | 1.329 | 235.430 | .195 | -.543 | 8.529 |
| | Low4_part pos | 3.198 | 1.617 | 219.603 | 1.000 | -2.325 | 8.721 |
| | Low4_pos | -1.034 | 1.457 | 238.517 | 1.000 | -6.007 | 3.938 |
| | other_neg | 3.494 | 1.047 | 252.593 | .064 | -.075 | 7.064 |
| | other_part pos | 2.421 | 1.081 | 250.977 | 1.000 | -1.266 | 6.108 |
| | other_pos | 2.331 | 1.318 | 247.957 | 1.000 | -2.165 | 6.828 |
| | PD_neg | 4.254* | 1.154 | 252.915 | .018 | .319 | 8.189 |
| | PD_part pos | 1.910 | 1.295 | 242.444 | 1.000 | -2.509 | 6.328 |

Based on estimated marginal means

*. The mean difference is significant at the .05 level.

a. Dependent Variable: DPDmax.

c. Adjustment for multiple comparisons: Bonferroni.

**Table S5:** Pairwise comparisons for two-way interaction model between hind limb diagnostic anaesthesia 'efficacy' and 'type' for outcome variable DPDmin, i.e. pelvic movement asymmetry associated with changes in weight-bearing between contra-lateral hind limbs.
Given are mean differences (in mm), standard error (in mm), degrees of freedom, significance achieved, upper and lower bounds of 95% confidence intervals for the pairwise difference (in mm).
Pairwise significant differences at P<0.05 after Bonferroni correction are indicated with '*'.
Forelimb block types: ASNB: abaxial sesamoid nerve block; DBLPN: deep branch of lateral plantar nerve block; Low6: Low6-point nerve block; MTPJ: metatarsophalangeal joint block; TMTJ: tarsometatarsal joint block; other: all other nerve/joint blocks.
Efficacy: neg: negative block (0 to 30% perceived change); part pos: partially positive block (>30 to 70% perceived change; pos: positive block (>70% change).

**Pairwise Comparisons[a]**

| (I) Type x Efficacy | (J) Type x Efficacy | Mean Difference (I-J) | Std. Error | df | Sig.[c] | 95% Confidence Interval for Difference[c] Lower Bound | Upper Bound |
|---|---|---|---|---|---|---|---|
| ASNB_neg | ASNB_part pos | 2.855 | 1.889 | 317.633 | 1.000 | -4.008 | 9.719 |
| | ASNB_pos | -1.529 | 2.580 | 298.813 | 1.000 | -10.907 | 7.848 |
| | DBLPN_neg | 2.674 | 1.646 | 309.600 | 1.000 | -3.308 | 8.655 |
| | DBLPN_part pos | 2.744 | 1.595 | 303.912 | 1.000 | -3.053 | 8.541 |
| | DBLPN_pos | 5.103 | 1.444 | 293.068 | .072 | -.146 | 10.352 |
| | Low6_neg | .191 | 1.462 | 301.580 | 1.000 | -5.124 | 5.505 |
| | Low6_part pos | 5.639 | 1.649 | 290.883 | .110 | -.357 | 11.636 |
| | Low6_pos | 5.564 | 1.620 | 284.607 | .104 | -.329 | 11.458 |
| | MTPJ_neg | -.289 | 1.846 | 312.721 | 1.000 | -6.995 | 6.417 |
| | MTPJ_part pos | 7.971* | 1.829 | 317.722 | .003 | 1.327 | 14.616 |
| | MTPJ_pos | 5.215 | 1.966 | 320.402 | 1.000 | -1.927 | 12.357 |
| | other_neg | .873 | 1.525 | 300.577 | 1.000 | -4.670 | 6.416 |
| | other_part pos | 4.995 | 1.514 | 302.180 | .166 | -.509 | 10.500 |
| | other_pos | 4.220 | 1.790 | 319.898 | 1.000 | -2.280 | 10.721 |
| | TMTJ_neg | 1.039 | 1.288 | 284.668 | 1.000 | -3.644 | 5.723 |
| | TMTJ_part pos | 1.869 | 1.655 | 323.678 | 1.000 | -4.143 | 7.881 |
| | TMTJ_pos | 4.157 | 1.561 | 321.066 | 1.000 | -1.514 | 9.828 |

| | | | | | | | |
|---|---|---|---|---|---|---|---|
| ASNB_part pos | ASNB_neg | -2.855 | 1.889 | 317.633 | 1.000 | -9.719 | 4.008 |
| | ASNB_pos | -4.385 | 2.755 | 313.849 | 1.000 | -14.393 | 5.623 |
| | DBLPN_neg | -.182 | 1.884 | 319.416 | 1.000 | -7.026 | 6.663 |
| | DBLPN_part pos | -.111 | 1.852 | 320.934 | 1.000 | -6.839 | 6.616 |
| | DBLPN_pos | 2.248 | 1.736 | 323.643 | 1.000 | -4.057 | 8.552 |
| | Low6_neg | -2.665 | 1.738 | 322.554 | 1.000 | -8.978 | 3.649 |
| | Low6_part pos | 2.784 | 1.890 | 309.811 | 1.000 | -4.086 | 9.654 |
| | Low6_pos | 2.709 | 1.892 | 320.868 | 1.000 | -4.165 | 9.582 |
| | MTPJ_neg | -3.144 | 2.002 | 278.720 | 1.000 | -10.429 | 4.141 |
| | MTPJ_part pos | 5.116 | 2.046 | 318.886 | 1.000 | -2.318 | 12.549 |
| | MTPJ_pos | 2.359 | 2.164 | 322.635 | 1.000 | -5.499 | 10.218 |
| | other_neg | -1.983 | 1.793 | 322.203 | 1.000 | -8.497 | 4.532 |
| | other_part pos | 2.140 | 1.756 | 305.790 | 1.000 | -4.243 | 8.523 |
| | other_pos | 1.365 | 2.014 | 317.357 | 1.000 | -5.953 | 8.683 |
| | TMTJ_neg | -1.816 | 1.608 | 323.894 | 1.000 | -7.657 | 4.024 |
| | TMTJ_part pos | -.987 | 1.891 | 323.992 | 1.000 | -7.854 | 5.880 |
| | TMTJ_pos | 1.301 | 1.786 | 323.021 | 1.000 | -5.186 | 7.789 |
| ASNB_pos | ASNB_neg | 1.529 | 2.580 | 298.813 | 1.000 | -7.848 | 10.907 |
| | ASNB_part pos | 4.385 | 2.755 | 313.849 | 1.000 | -5.623 | 14.393 |
| | DBLPN_neg | 4.203 | 2.596 | 310.720 | 1.000 | -5.232 | 13.638 |
| | DBLPN_part pos | 4.273 | 2.573 | 312.043 | 1.000 | -5.075 | 13.622 |
| | DBLPN_pos | 6.633 | 2.483 | 310.846 | 1.000 | -2.390 | 15.655 |
| | Low6_neg | 1.720 | 2.486 | 310.219 | 1.000 | -7.314 | 10.754 |
| | Low6_part pos | 7.169 | 2.609 | 308.902 | .973 | -2.313 | 16.651 |
| | Low6_pos | 7.093 | 2.542 | 277.770 | .861 | -2.155 | 16.342 |
| | MTPJ_neg | 1.240 | 2.731 | 312.745 | 1.000 | -8.684 | 11.165 |
| | MTPJ_part pos | 9.501 | 2.722 | 323.759 | .084 | -.385 | 19.387 |
| | MTPJ_pos | 6.744 | 2.822 | 318.444 | 1.000 | -3.506 | 16.995 |
| | other_neg | 2.402 | 2.521 | 307.519 | 1.000 | -6.759 | 11.564 |

| | | | | | | | |
|---|---|---|---|---|---|---|---|
| | other_part pos | 6.525 | 2.508 | 300.991 | 1.000 | -2.590 | 15.640 |
| | other_pos | 5.750 | 2.685 | 321.970 | 1.000 | -4.004 | 15.504 |
| | TMTJ_neg | 2.568 | 2.381 | 302.736 | 1.000 | -6.087 | 11.224 |
| | TMTJ_part pos | 3.398 | 2.602 | 318.538 | 1.000 | -6.055 | 12.851 |
| | TMTJ_pos | 5.686 | 2.540 | 320.632 | 1.000 | -3.539 | 14.911 |
| DBLPN_neg | ASNB_neg | -2.674 | 1.646 | 309.600 | 1.000 | -8.655 | 3.308 |
| | ASNB_part pos | .182 | 1.884 | 319.416 | 1.000 | -6.663 | 7.026 |
| | ASNB_pos | -4.203 | 2.596 | 310.720 | 1.000 | -13.638 | 5.232 |
| | DBLPN_part pos | .070 | 1.617 | 321.737 | 1.000 | -5.804 | 5.945 |
| | DBLPN_pos | 2.429 | 1.471 | 321.893 | 1.000 | -2.914 | 7.773 |
| | Low6_neg | -2.483 | 1.437 | 280.428 | 1.000 | -7.710 | 2.744 |
| | Low6_part pos | 2.966 | 1.671 | 315.691 | 1.000 | -3.104 | 9.035 |
| | Low6_pos | 2.890 | 1.659 | 320.716 | 1.000 | -3.136 | 8.917 |
| | MTPJ_neg | -2.963 | 1.862 | 322.208 | 1.000 | -9.724 | 3.799 |
| | MTPJ_part pos | 5.298 | 1.845 | 304.450 | .669 | -1.407 | 12.003 |
| | MTPJ_pos | 2.541 | 1.997 | 322.052 | 1.000 | -4.713 | 9.795 |
| | other_neg | -1.801 | 1.545 | 321.502 | 1.000 | -7.412 | 3.810 |
| | other_part pos | 2.322 | 1.521 | 315.474 | 1.000 | -3.205 | 7.848 |
| | other_pos | 1.547 | 1.779 | 319.991 | 1.000 | -4.916 | 8.009 |
| | TMTJ_neg | -1.635 | 1.294 | 307.659 | 1.000 | -6.338 | 3.069 |
| | TMTJ_part pos | -.805 | 1.664 | 322.338 | 1.000 | -6.849 | 5.238 |
| | TMTJ_pos | 1.483 | 1.550 | 322.872 | 1.000 | -4.147 | 7.113 |
| DBLPN_part pos | ASNB_neg | -2.744 | 1.595 | 303.912 | 1.000 | -8.541 | 3.053 |
| | ASNB_part pos | .111 | 1.852 | 320.934 | 1.000 | -6.616 | 6.839 |
| | ASNB_pos | -4.273 | 2.573 | 312.043 | 1.000 | -13.622 | 5.075 |
| | DBLPN_neg | -.070 | 1.617 | 321.737 | 1.000 | -5.945 | 5.804 |
| | DBLPN_pos | 2.359 | 1.416 | 318.630 | 1.000 | -2.785 | 7.504 |
| | Low6_neg | -2.553 | 1.413 | 307.563 | 1.000 | -7.689 | 2.582 |
| | Low6_part pos | 2.895 | 1.594 | 285.032 | 1.000 | -2.902 | 8.692 |

| | | | | | | | |
|---|---|---|---|---|---|---|---|
| | Low6_pos | 2.820 | 1.618 | 320.039 | 1.000 | -3.059 | 8.699 |
| | MTPJ_neg | -3.033 | 1.795 | 313.127 | 1.000 | -9.553 | 3.487 |
| | MTPJ_part pos | 5.227 | 1.811 | 298.908 | .640 | -1.357 | 11.811 |
| | MTPJ_pos | 2.471 | 1.965 | 321.260 | 1.000 | -4.667 | 9.608 |
| | other_neg | -1.871 | 1.511 | 324.000 | 1.000 | -7.361 | 3.619 |
| | other_part pos | 2.251 | 1.475 | 316.017 | 1.000 | -3.109 | 7.612 |
| | other_pos | 1.476 | 1.758 | 309.378 | 1.000 | -4.914 | 7.866 |
| | TMTJ_neg | -1.705 | 1.256 | 320.556 | 1.000 | -6.268 | 2.858 |
| | TMTJ_part pos | -.875 | 1.612 | 323.706 | 1.000 | -6.730 | 4.979 |
| | TMTJ_pos | 1.413 | 1.518 | 315.669 | 1.000 | -4.101 | 6.926 |
| DBLPN_pos | ASNB_neg | -5.103 | 1.444 | 293.068 | .072 | -10.352 | .146 |
| | ASNB_part pos | -2.248 | 1.736 | 323.643 | 1.000 | -8.552 | 4.057 |
| | ASNB_pos | -6.633 | 2.483 | 310.846 | 1.000 | -15.655 | 2.390 |
| | DBLPN_neg | -2.429 | 1.471 | 321.893 | 1.000 | -7.773 | 2.914 |
| | DBLPN_part pos | -2.359 | 1.416 | 318.630 | 1.000 | -7.504 | 2.785 |
| | Low6_neg | -4.912* | 1.239 | 298.677 | .014 | -9.418 | -.407 |
| | Low6_part pos | .536 | 1.475 | 305.174 | 1.000 | -4.826 | 5.898 |
| | Low6_pos | .461 | 1.464 | 315.595 | 1.000 | -4.856 | 5.778 |
| | MTPJ_neg | -5.392 | 1.699 | 322.900 | .252 | -11.562 | .778 |
| | MTPJ_part pos | 2.868 | 1.682 | 287.573 | 1.000 | -3.250 | 8.986 |
| | MTPJ_pos | .112 | 1.841 | 321.020 | 1.000 | -6.577 | 6.800 |
| | other_neg | -4.230 | 1.346 | 323.784 | .280 | -9.120 | .659 |
| | other_part pos | -.108 | 1.336 | 323.744 | 1.000 | -4.960 | 4.745 |
| | other_pos | -.883 | 1.611 | 312.303 | 1.000 | -6.736 | 4.970 |
| | TMTJ_neg | -4.064* | 1.059 | 316.302 | .023 | -7.911 | -.217 |
| | TMTJ_part pos | -3.235 | 1.453 | 323.116 | 1.000 | -8.514 | 2.044 |
| | TMTJ_pos | -.947 | 1.367 | 298.930 | 1.000 | -5.918 | 4.025 |
| Low6_neg | ASNB_neg | -.191 | 1.462 | 301.580 | 1.000 | -5.505 | 5.124 |
| | ASNB_part pos | 2.665 | 1.738 | 322.554 | 1.000 | -3.649 | 8.978 |

| | | | | | | | |
|---|---|---|---|---|---|---|---|
| | ASNB_pos | -1.720 | 2.486 | 310.219 | 1.000 | -10.754 | 7.314 |
| | DBLPN_neg | 2.483 | 1.437 | 280.428 | 1.000 | -2.744 | 7.710 |
| | DBLPN_part pos | 2.553 | 1.413 | 307.563 | 1.000 | -2.582 | 7.689 |
| | DBLPN_pos | 4.912* | 1.239 | 298.677 | .014 | .407 | 9.418 |
| | Low6_part pos | 5.449* | 1.496 | 313.950 | .048 | .014 | 10.883 |
| | Low6_pos | 5.373 | 1.485 | 322.057 | .053 | -.020 | 10.767 |
| | MTPJ_neg | -.480 | 1.703 | 322.271 | 1.000 | -6.666 | 5.707 |
| | MTPJ_part pos | 7.781* | 1.669 | 307.640 | .001 | 1.717 | 13.844 |
| | MTPJ_pos | 5.024 | 1.851 | 319.966 | 1.000 | -1.701 | 11.749 |
| | other_neg | .682 | 1.355 | 323.430 | 1.000 | -4.241 | 5.605 |
| | other_part pos | 4.805 | 1.343 | 322.999 | .061 | -.073 | 9.682 |
| | other_pos | 4.030 | 1.619 | 312.629 | 1.000 | -1.852 | 9.912 |
| | TMTJ_neg | .848 | 1.059 | 299.353 | 1.000 | -3.000 | 4.696 |
| | TMTJ_part pos | 1.678 | 1.469 | 323.976 | 1.000 | -3.657 | 7.013 |
| | TMTJ_pos | 3.966 | 1.358 | 318.844 | .572 | -.966 | 8.898 |
| Low6_part pos | ASNB_neg | -5.639 | 1.649 | 290.883 | .110 | -11.636 | .357 |
| | ASNB_part pos | -2.784 | 1.890 | 309.811 | 1.000 | -9.654 | 4.086 |
| | ASNB_pos | -7.169 | 2.609 | 308.902 | .973 | -16.651 | 2.313 |
| | DBLPN_neg | -2.966 | 1.671 | 315.691 | 1.000 | -9.035 | 3.104 |
| | DBLPN_part pos | -2.895 | 1.594 | 285.032 | 1.000 | -8.692 | 2.902 |
| | DBLPN_pos | -.536 | 1.475 | 305.174 | 1.000 | -5.898 | 4.826 |
| | Low6_neg | -5.449* | 1.496 | 313.950 | .048 | -10.883 | -.014 |
| | Low6_pos | -.075 | 1.677 | 315.810 | 1.000 | -6.168 | 6.017 |
| | MTPJ_neg | -5.928 | 1.851 | 307.583 | .230 | -12.655 | .798 |
| | MTPJ_part pos | 2.332 | 1.846 | 318.972 | 1.000 | -4.373 | 9.037 |
| | MTPJ_pos | -.425 | 2.003 | 323.999 | 1.000 | -7.701 | 6.852 |
| | other_neg | -4.767 | 1.567 | 319.314 | .388 | -10.457 | .924 |
| | other_part pos | -.644 | 1.540 | 309.617 | 1.000 | -6.239 | 4.951 |
| | other_pos | -1.419 | 1.811 | 317.628 | 1.000 | -7.999 | 5.161 |

| | | | | | | | |
|---|---|---|---|---|---|---|---|
| | TMTJ_neg | -4.600 | 1.330 | 310.831 | .094 | -9.432 | .231 |
| | TMTJ_part pos | -3.771 | 1.663 | 320.850 | 1.000 | -9.812 | 2.271 |
| | TMTJ_pos | -1.483 | 1.584 | 320.598 | 1.000 | -7.236 | 4.270 |
| Low6_pos | ASNB_neg | -5.564 | 1.620 | 284.607 | .104 | -11.458 | .329 |
| | ASNB_part pos | -2.709 | 1.892 | 320.868 | 1.000 | -9.582 | 4.165 |
| | ASNB_pos | -7.093 | 2.542 | 277.770 | .861 | -16.342 | 2.155 |
| | DBLPN_neg | -2.890 | 1.659 | 320.716 | 1.000 | -8.917 | 3.136 |
| | DBLPN_part pos | -2.820 | 1.618 | 320.039 | 1.000 | -8.699 | 3.059 |
| | DBLPN_pos | -.461 | 1.464 | 315.595 | 1.000 | -5.778 | 4.856 |
| | Low6_neg | -5.373 | 1.485 | 322.057 | .053 | -10.767 | .020 |
| | Low6_part pos | .075 | 1.677 | 315.810 | 1.000 | -6.017 | 6.168 |
| | MTPJ_neg | -5.853 | 1.852 | 317.269 | .264 | -12.580 | .874 |
| | MTPJ_part pos | 2.407 | 1.845 | 306.871 | 1.000 | -4.299 | 9.114 |
| | MTPJ_pos | -.349 | 1.938 | 308.277 | 1.000 | -7.394 | 6.695 |
| | other_neg | -4.691 | 1.549 | 321.213 | .406 | -10.317 | .934 |
| | other_part pos | -.569 | 1.533 | 318.545 | 1.000 | -6.139 | 5.001 |
| | other_pos | -1.344 | 1.779 | 321.706 | 1.000 | -7.804 | 5.117 |
| | TMTJ_neg | -4.525 | 1.318 | 321.637 | .104 | -9.314 | .264 |
| | TMTJ_part pos | -3.695 | 1.660 | 323.830 | 1.000 | -9.724 | 2.333 |
| | TMTJ_pos | -1.407 | 1.573 | 311.935 | 1.000 | -7.124 | 4.309 |
| MTPJ_neg | ASNB_neg | .289 | 1.846 | 312.721 | 1.000 | -6.417 | 6.995 |
| | ASNB_part pos | 3.144 | 2.002 | 278.720 | 1.000 | -4.141 | 10.429 |
| | ASNB_pos | -1.240 | 2.731 | 312.745 | 1.000 | -11.165 | 8.684 |
| | DBLPN_neg | 2.963 | 1.862 | 322.208 | 1.000 | -3.799 | 9.724 |
| | DBLPN_part pos | 3.033 | 1.795 | 313.127 | 1.000 | -3.487 | 9.553 |
| | DBLPN_pos | 5.392 | 1.699 | 322.900 | .252 | -.778 | 11.562 |
| | Low6_neg | .480 | 1.703 | 322.271 | 1.000 | -5.707 | 6.666 |
| | Low6_part pos | 5.928 | 1.851 | 307.583 | .230 | -.798 | 12.655 |
| | Low6_pos | 5.853 | 1.852 | 317.269 | .264 | -.874 | 12.580 |

| | | | | | | | |
|---|---|---|---|---|---|---|---|
| | MTPJ_part pos | 8.260* | 2.032 | 309.158 | .009 | .875 | 15.645 |
| | MTPJ_pos | 5.504 | 2.167 | 323.552 | 1.000 | -2.366 | 13.373 |
| | other_neg | 1.162 | 1.732 | 313.627 | 1.000 | -5.133 | 7.456 |
| | other_part pos | 5.284 | 1.728 | 313.494 | .370 | -.994 | 11.563 |
| | other_pos | 4.509 | 1.985 | 316.047 | 1.000 | -2.703 | 11.722 |
| | TMTJ_neg | 1.328 | 1.557 | 322.025 | 1.000 | -4.328 | 6.985 |
| | TMTJ_part pos | 2.158 | 1.811 | 310.108 | 1.000 | -4.422 | 8.737 |
| | TMTJ_pos | 4.446 | 1.779 | 319.106 | 1.000 | -2.018 | 10.910 |
| MTPJ_part pos | ASNB_neg | -7.971* | 1.829 | 317.722 | .003 | -14.616 | -1.327 |
| | ASNB_part pos | -5.116 | 2.046 | 318.886 | 1.000 | -12.549 | 2.318 |
| | ASNB_pos | -9.501 | 2.722 | 323.759 | .084 | -19.387 | .385 |
| | DBLPN_neg | -5.298 | 1.845 | 304.450 | .669 | -12.003 | 1.407 |
| | DBLPN_part pos | -5.227 | 1.811 | 298.908 | .640 | -11.811 | 1.357 |
| | DBLPN_pos | -2.868 | 1.682 | 287.573 | 1.000 | -8.986 | 3.250 |
| | Low6_neg | -7.781* | 1.669 | 307.640 | .001 | -13.844 | -1.717 |
| | Low6_part pos | -2.332 | 1.846 | 318.972 | 1.000 | -9.037 | 4.373 |
| | Low6_pos | -2.407 | 1.845 | 306.871 | 1.000 | -9.114 | 4.299 |
| | MTPJ_neg | -8.260* | 2.032 | 309.158 | .009 | -15.645 | -.875 |
| | MTPJ_pos | -2.757 | 2.126 | 316.983 | 1.000 | -10.482 | 4.968 |
| | other_neg | -7.098* | 1.757 | 283.994 | .011 | -13.490 | -.707 |
| | other_part pos | -2.976 | 1.736 | 295.433 | 1.000 | -9.288 | 3.337 |
| | other_pos | -3.751 | 1.970 | 269.022 | 1.000 | -10.920 | 3.419 |
| | TMTJ_neg | -6.932* | 1.543 | 279.664 | .002 | -12.545 | -1.319 |
| | TMTJ_part pos | -6.103 | 1.853 | 279.790 | .171 | -12.843 | .638 |
| | TMTJ_pos | -3.815 | 1.762 | 261.119 | 1.000 | -10.231 | 2.601 |
| MTPJ_pos | ASNB_neg | -5.215 | 1.966 | 320.402 | 1.000 | -12.357 | 1.927 |
| | ASNB_part pos | -2.359 | 2.164 | 322.635 | 1.000 | -10.218 | 5.499 |
| | ASNB_pos | -6.744 | 2.822 | 318.444 | 1.000 | -16.995 | 3.506 |
| | DBLPN_neg | -2.541 | 1.997 | 322.052 | 1.000 | -9.795 | 4.713 |

| | | | | | | | |
|---|---|---|---|---|---|---|---|
| | DBLPN_part pos | -2.471 | 1.965 | 321.260 | 1.000 | -9.608 | 4.667 |
| | DBLPN_pos | -.112 | 1.841 | 321.020 | 1.000 | -6.800 | 6.577 |
| | Low6_neg | -5.024 | 1.851 | 319.966 | 1.000 | -11.749 | 1.701 |
| | Low6_part pos | .425 | 2.003 | 323.999 | 1.000 | -6.852 | 7.701 |
| | Low6_pos | .349 | 1.938 | 308.277 | 1.000 | -6.695 | 7.394 |
| | MTPJ_neg | -5.504 | 2.167 | 323.552 | 1.000 | -13.373 | 2.366 |
| | MTPJ_part pos | 2.757 | 2.126 | 316.983 | 1.000 | -4.968 | 10.482 |
| | other_neg | -4.342 | 1.896 | 322.807 | 1.000 | -11.227 | 2.543 |
| | other_part pos | -.219 | 1.860 | 323.010 | 1.000 | -6.975 | 6.537 |
| | other_pos | -.994 | 2.110 | 305.709 | 1.000 | -8.664 | 6.676 |
| | TMTJ_neg | -4.176 | 1.730 | 314.343 | 1.000 | -10.462 | 2.111 |
| | TMTJ_part pos | -3.346 | 2.003 | 312.374 | 1.000 | -10.626 | 3.933 |
| | TMTJ_pos | -1.058 | 1.920 | 303.907 | 1.000 | -8.038 | 5.922 |
| other_neg | ASNB_neg | -.873 | 1.525 | 300.577 | 1.000 | -6.416 | 4.670 |
| | ASNB_part pos | 1.983 | 1.793 | 322.203 | 1.000 | -4.532 | 8.497 |
| | ASNB_pos | -2.402 | 2.521 | 307.519 | 1.000 | -11.564 | 6.759 |
| | DBLPN_neg | 1.801 | 1.545 | 321.502 | 1.000 | -3.810 | 7.412 |
| | DBLPN_part pos | 1.871 | 1.511 | 324.000 | 1.000 | -3.619 | 7.361 |
| | DBLPN_pos | 4.230 | 1.346 | 323.784 | .280 | -.659 | 9.120 |
| | Low6_neg | -.682 | 1.355 | 323.430 | 1.000 | -5.605 | 4.241 |
| | Low6_part pos | 4.767 | 1.567 | 319.314 | .388 | -.924 | 10.457 |
| | Low6_pos | 4.691 | 1.549 | 321.213 | .406 | -.934 | 10.317 |
| | MTPJ_neg | -1.162 | 1.732 | 313.627 | 1.000 | -7.456 | 5.133 |
| | MTPJ_part pos | 7.098* | 1.757 | 283.994 | .011 | .707 | 13.490 |
| | MTPJ_pos | 4.342 | 1.896 | 322.807 | 1.000 | -2.543 | 11.227 |
| | other_part pos | 4.123 | 1.387 | 307.141 | .488 | -.918 | 9.163 |
| | other_pos | 3.348 | 1.696 | 304.125 | 1.000 | -2.815 | 9.510 |
| | TMTJ_neg | .166 | 1.184 | 322.294 | 1.000 | -4.132 | 4.465 |
| | TMTJ_part pos | .996 | 1.560 | 316.039 | 1.000 | -4.671 | 6.663 |

| | | | | | | | |
|---|---|---|---|---|---|---|---|
| | TMTJ_pos | 3.284 | 1.458 | 295.245 | 1.000 | -2.017 | 8.585 |
| other_part pos | ASNB_neg | -4.995 | 1.514 | 302.180 | .166 | -10.500 | .509 |
| | ASNB_part pos | -2.140 | 1.756 | 305.790 | 1.000 | -8.523 | 4.243 |
| | ASNB_pos | -6.525 | 2.508 | 300.991 | 1.000 | -15.640 | 2.590 |
| | DBLPN_neg | -2.322 | 1.521 | 315.474 | 1.000 | -7.848 | 3.205 |
| | DBLPN_part pos | -2.251 | 1.475 | 316.017 | 1.000 | -7.612 | 3.109 |
| | DBLPN_pos | .108 | 1.336 | 323.744 | 1.000 | -4.745 | 4.960 |
| | Low6_neg | -4.805 | 1.343 | 322.999 | .061 | -9.682 | .073 |
| | Low6_part pos | .644 | 1.540 | 309.617 | 1.000 | -4.951 | 6.239 |
| | Low6_pos | .569 | 1.533 | 318.545 | 1.000 | -5.001 | 6.139 |
| | MTPJ_neg | -5.284 | 1.728 | 313.494 | .370 | -11.563 | .994 |
| | MTPJ_part pos | 2.976 | 1.736 | 295.433 | 1.000 | -3.337 | 9.288 |
| | MTPJ_pos | .219 | 1.860 | 323.010 | 1.000 | -6.537 | 6.975 |
| | other_neg | -4.123 | 1.387 | 307.141 | .488 | -9.163 | .918 |
| | other_pos | -.775 | 1.661 | 320.583 | 1.000 | -6.809 | 5.259 |
| | TMTJ_neg | -3.956 | 1.156 | 323.103 | .107 | -8.156 | .243 |
| | TMTJ_part pos | -3.127 | 1.541 | 320.080 | 1.000 | -8.724 | 2.471 |
| | TMTJ_pos | -.839 | 1.424 | 318.444 | 1.000 | -6.012 | 4.335 |
| other_pos | ASNB_neg | -4.220 | 1.790 | 319.898 | 1.000 | -10.721 | 2.280 |
| | ASNB_part pos | -1.365 | 2.014 | 317.357 | 1.000 | -8.683 | 5.953 |
| | ASNB_pos | -5.750 | 2.685 | 321.970 | 1.000 | -15.504 | 4.004 |
| | DBLPN_neg | -1.547 | 1.779 | 319.991 | 1.000 | -8.009 | 4.916 |
| | DBLPN_part pos | -1.476 | 1.758 | 309.378 | 1.000 | -7.866 | 4.914 |
| | DBLPN_pos | .883 | 1.611 | 312.303 | 1.000 | -4.970 | 6.736 |
| | Low6_neg | -4.030 | 1.619 | 312.629 | 1.000 | -9.912 | 1.852 |
| | Low6_part pos | 1.419 | 1.811 | 317.628 | 1.000 | -5.161 | 7.999 |
| | Low6_pos | 1.344 | 1.779 | 321.706 | 1.000 | -5.117 | 7.804 |
| | MTPJ_neg | -4.509 | 1.985 | 316.047 | 1.000 | -11.722 | 2.703 |
| | MTPJ_part pos | 3.751 | 1.970 | 269.022 | 1.000 | -3.419 | 10.920 |

| | | | | | | | |
|---|---|---|---|---|---|---|---|
| | MTPJ_pos | .994 | 2.110 | 305.709 | 1.000 | -6.676 | 8.664 |
| | other_neg | -3.348 | 1.696 | 304.125 | 1.000 | -9.510 | 2.815 |
| | other_part pos | .775 | 1.661 | 320.583 | 1.000 | -5.259 | 6.809 |
| | TMTJ_neg | -3.181 | 1.482 | 293.300 | 1.000 | -8.572 | 2.209 |
| | TMTJ_part pos | -2.352 | 1.800 | 292.618 | 1.000 | -8.898 | 4.195 |
| | TMTJ_pos | -.064 | 1.707 | 275.570 | 1.000 | -6.275 | 6.148 |
| TMTJ_neg | ASNB_neg | -1.039 | 1.288 | 284.668 | 1.000 | -5.723 | 3.644 |
| | ASNB_part pos | 1.816 | 1.608 | 323.894 | 1.000 | -4.024 | 7.657 |
| | ASNB_pos | -2.568 | 2.381 | 302.736 | 1.000 | -11.224 | 6.087 |
| | DBLPN_neg | 1.635 | 1.294 | 307.659 | 1.000 | -3.069 | 6.338 |
| | DBLPN_part pos | 1.705 | 1.256 | 320.556 | 1.000 | -2.858 | 6.268 |
| | DBLPN_pos | 4.064* | 1.059 | 316.302 | .023 | .217 | 7.911 |
| | Low6_neg | -.848 | 1.059 | 299.353 | 1.000 | -4.696 | 3.000 |
| | Low6_part pos | 4.600 | 1.330 | 310.831 | .094 | -.231 | 9.432 |
| | Low6_pos | 4.525 | 1.318 | 321.637 | .104 | -.264 | 9.314 |
| | MTPJ_neg | -1.328 | 1.557 | 322.025 | 1.000 | -6.985 | 4.328 |
| | MTPJ_part pos | 6.932* | 1.543 | 279.664 | .002 | 1.319 | 12.545 |
| | MTPJ_pos | 4.176 | 1.730 | 314.343 | 1.000 | -2.111 | 10.462 |
| | other_neg | -.166 | 1.184 | 322.294 | 1.000 | -4.465 | 4.132 |
| | other_part pos | 3.956 | 1.156 | 323.103 | .107 | -.243 | 8.156 |
| | other_pos | 3.181 | 1.482 | 293.300 | 1.000 | -2.209 | 8.572 |
| | TMTJ_part pos | .829 | 1.319 | 317.785 | 1.000 | -3.963 | 5.622 |
| | TMTJ_pos | 3.118 | 1.203 | 274.282 | 1.000 | -1.261 | 7.496 |
| TMTJ_part pos | ASNB_neg | -1.869 | 1.655 | 323.678 | 1.000 | -7.881 | 4.143 |
| | ASNB_part pos | .987 | 1.891 | 323.992 | 1.000 | -5.880 | 7.854 |
| | ASNB_pos | -3.398 | 2.602 | 318.538 | 1.000 | -12.851 | 6.055 |
| | DBLPN_neg | .805 | 1.664 | 322.338 | 1.000 | -5.238 | 6.849 |
| | DBLPN_part pos | .875 | 1.612 | 323.706 | 1.000 | -4.979 | 6.730 |
| | DBLPN_pos | 3.235 | 1.453 | 323.116 | 1.000 | -2.044 | 8.514 |

| | | | | | | | |
|---|---|---|---|---|---|---|---|
| | Low6_neg | -1.678 | 1.469 | 323.976 | 1.000 | -7.013 | 3.657 |
| | Low6_part pos | 3.771 | 1.663 | 320.850 | 1.000 | -2.271 | 9.812 |
| | Low6_pos | 3.695 | 1.660 | 323.830 | 1.000 | -2.333 | 9.724 |
| | MTPJ_neg | -2.158 | 1.811 | 310.108 | 1.000 | -8.737 | 4.422 |
| | MTPJ_part pos | 6.103 | 1.853 | 279.790 | .171 | -.638 | 12.843 |
| | MTPJ_pos | 3.346 | 2.003 | 312.374 | 1.000 | -3.933 | 10.626 |
| | other_neg | -.996 | 1.560 | 316.039 | 1.000 | -6.663 | 4.671 |
| | other_part pos | 3.127 | 1.541 | 320.080 | 1.000 | -2.471 | 8.724 |
| | other_pos | 2.352 | 1.800 | 292.618 | 1.000 | -4.195 | 8.898 |
| | TMTJ_neg | -.829 | 1.319 | 317.785 | 1.000 | -5.622 | 3.963 |
| | TMTJ_pos | 2.288 | 1.572 | 288.229 | 1.000 | -3.428 | 8.004 |
| TMTJ_pos | ASNB_neg | -4.157 | 1.561 | 321.066 | 1.000 | -9.828 | 1.514 |
| | ASNB_part pos | -1.301 | 1.786 | 323.021 | 1.000 | -7.789 | 5.186 |
| | ASNB_pos | -5.686 | 2.540 | 320.632 | 1.000 | -14.911 | 3.539 |
| | DBLPN_neg | -1.483 | 1.550 | 322.872 | 1.000 | -7.113 | 4.147 |
| | DBLPN_part pos | -1.413 | 1.518 | 315.669 | 1.000 | -6.926 | 4.101 |
| | DBLPN_pos | .947 | 1.367 | 298.930 | 1.000 | -4.025 | 5.918 |
| | Low6_neg | -3.966 | 1.358 | 318.844 | .572 | -8.898 | .966 |
| | Low6_part pos | 1.483 | 1.584 | 320.598 | 1.000 | -4.270 | 7.236 |
| | Low6_pos | 1.407 | 1.573 | 311.935 | 1.000 | -4.309 | 7.124 |
| | MTPJ_neg | -4.446 | 1.779 | 319.106 | 1.000 | -10.910 | 2.018 |
| | MTPJ_part pos | 3.815 | 1.762 | 261.119 | 1.000 | -2.601 | 10.231 |
| | MTPJ_pos | 1.058 | 1.920 | 303.907 | 1.000 | -5.922 | 8.038 |
| | other_neg | -3.284 | 1.458 | 295.245 | 1.000 | -8.585 | 2.017 |
| | other_part pos | .839 | 1.424 | 318.444 | 1.000 | -4.335 | 6.012 |
| | other_pos | .064 | 1.707 | 275.570 | 1.000 | -6.148 | 6.275 |
| | TMTJ_neg | -3.118 | 1.203 | 274.282 | 1.000 | -7.496 | 1.261 |
| | TMTJ_part pos | -2.288 | 1.572 | 288.229 | 1.000 | -8.004 | 3.428 |

Based on estimated marginal means

*. The mean difference is significant at the .05 level.

a. Dependent Variable: DPDmin.

c. Adjustment for multiple comparisons: Bonferroni.

**Table S6:** Pairwise comparisons for two-way interaction model between hind limb diagnostic anaesthesia 'efficacy' and 'type' for outcome variable DPDup, i.e. pelvic movement asymmetry associated with changes in upward movement amplitudes between stride halves.
Given are mean differences (in mm), standard error (in mm), degrees of freedom, significance achieved, upper and lower bounds of 95% confidence intervals for the pairwise difference (in mm). Pairwise significant differences at P<0.05 after Bonferroni correction are indicated with '*'.
Forelimb block types: ASNB: abaxial sesamoid nerve block; DBLPN: deep branch of lateral plantar nerve block; Low6: Low6-point nerve block; MTPJ: metatarsophalangeal joint block; TMTJ: tarsometatarsal joint block; other: all other nerve/joint blocks.
Efficacy: neg: negative block (0 to 30% perceived change); part pos: partially positive block (>30 to 70% perceived change; pos: positive block (>70% change).

**Pairwise Comparisons[a]**

| (I) Type x Efficacy | (J) Type x Efficacy | Mean Difference (I-J) | Std. Error | df | Sig.[c] | 95% Confidence Interval for Difference[c] Lower Bound | Upper Bound |
|---|---|---|---|---|---|---|---|
| ASNB_neg | ASNB_part pos | 6.968 | 2.910 | 323.957 | 1.000 | -3.603 | 17.539 |
| | ASNB_pos | .977 | 3.997 | 317.630 | 1.000 | -13.545 | 15.499 |
| | DBLPN_neg | 4.068 | 2.543 | 322.426 | 1.000 | -5.169 | 13.304 |
| | DBLPN_part pos | 7.405 | 2.468 | 320.858 | .444 | -1.559 | 16.369 |
| | DBLPN_pos | 8.837* | 2.240 | 315.620 | .015 | .700 | 16.974 |
| | Low6_neg | .559 | 2.263 | 320.067 | 1.000 | -7.662 | 8.781 |
| | Low6_part pos | 9.438* | 2.561 | 312.436 | .041 | .133 | 18.743 |
| | Low6_pos | 9.983* | 2.519 | 310.813 | .014 | .830 | 19.135 |
| | MTPJ_neg | -1.768 | 2.845 | 323.950 | 1.000 | -12.100 | 8.564 |
| | MTPJ_part pos | 11.805* | 2.789 | 312.596 | .005 | 1.671 | 21.939 |
| | MTPJ_pos | 6.986 | 3.024 | 323.597 | 1.000 | -3.996 | 17.968 |
| | other_neg | .568 | 2.361 | 320.320 | 1.000 | -8.007 | 9.143 |
| | other_part pos | 9.764* | 2.343 | 321.708 | .006 | 1.254 | 18.273 |
| | other_pos | 9.741 | 2.731 | 313.393 | .064 | -.181 | 19.664 |
| | TMTJ_neg | .181 | 2.001 | 313.602 | 1.000 | -7.088 | 7.449 |
| | TMTJ_part pos | 1.014 | 2.538 | 320.424 | 1.000 | -8.204 | 10.233 |
| | TMTJ_pos | 7.111 | 2.387 | 319.224 | .476 | -1.559 | 15.781 |
| ASNB_part pos | ASNB_neg | -6.968 | 2.910 | 323.957 | 1.000 | -17.539 | 3.603 |

| | | | | | | | |
|---|---|---|---|---|---|---|---|
| | ASNB_pos | -5.991 | 4.251 | 322.831 | 1.000 | -21.432 | 9.451 |
| | DBLPN_neg | -2.900 | 2.902 | 323.810 | 1.000 | -13.442 | 7.641 |
| | DBLPN_part pos | .437 | 2.849 | 323.974 | 1.000 | -9.912 | 10.786 |
| | DBLPN_pos | 1.869 | 2.665 | 323.281 | 1.000 | -7.810 | 11.548 |
| | Low6_neg | -6.408 | 2.672 | 323.892 | 1.000 | -16.114 | 3.297 |
| | Low6_part pos | 2.470 | 2.923 | 320.332 | 1.000 | -8.148 | 13.088 |
| | Low6_pos | 3.015 | 2.911 | 323.973 | 1.000 | -7.559 | 13.589 |
| | MTPJ_neg | -8.735 | 3.116 | 309.217 | .822 | -20.057 | 2.586 |
| | MTPJ_part pos | 4.837 | 3.123 | 314.565 | 1.000 | -6.508 | 16.182 |
| | MTPJ_pos | .018 | 3.324 | 323.605 | 1.000 | -12.056 | 12.093 |
| | other_neg | -6.400 | 2.757 | 323.795 | 1.000 | -16.413 | 3.614 |
| | other_part pos | 2.796 | 2.717 | 320.610 | 1.000 | -7.074 | 12.665 |
| | other_pos | 2.774 | 3.072 | 313.038 | 1.000 | -8.387 | 13.934 |
| | TMTJ_neg | -6.787 | 2.467 | 322.534 | .959 | -15.747 | 2.173 |
| | TMTJ_part pos | -5.953 | 2.897 | 320.750 | 1.000 | -16.476 | 4.569 |
| | TMTJ_pos | .143 | 2.746 | 324.000 | 1.000 | -9.832 | 10.118 |
| ASNB_pos | ASNB_neg | -.977 | 3.997 | 317.630 | 1.000 | -15.499 | 13.545 |
| | ASNB_part pos | 5.991 | 4.251 | 322.831 | 1.000 | -9.451 | 21.432 |
| | DBLPN_neg | 3.090 | 4.012 | 321.844 | 1.000 | -11.481 | 17.662 |
| | DBLPN_part pos | 6.428 | 3.973 | 322.584 | 1.000 | -8.003 | 20.859 |
| | DBLPN_pos | 7.860 | 3.836 | 321.930 | 1.000 | -6.074 | 21.794 |
| | Low6_neg | -.418 | 3.842 | 321.509 | 1.000 | -14.373 | 13.538 |
| | Low6_part pos | 8.461 | 4.034 | 320.975 | 1.000 | -6.192 | 23.114 |
| | Low6_pos | 9.006 | 3.959 | 302.950 | 1.000 | -5.386 | 23.397 |
| | MTPJ_neg | -2.745 | 4.214 | 323.653 | 1.000 | -18.051 | 12.561 |
| | MTPJ_part pos | 10.828 | 4.177 | 323.107 | 1.000 | -4.345 | 26.001 |
| | MTPJ_pos | 6.009 | 4.345 | 323.998 | 1.000 | -9.772 | 21.790 |
| | other_neg | -.409 | 3.898 | 320.985 | 1.000 | -14.568 | 13.751 |
| | other_part pos | 8.787 | 3.883 | 319.522 | 1.000 | -5.318 | 22.891 |

| | | | | | | | |
|---|---|---|---|---|---|---|---|
| | other_pos | 8.764 | 4.129 | 323.923 | 1.000 | -6.234 | 23.762 |
| | TMTJ_neg | -.796 | 3.687 | 318.591 | 1.000 | -14.192 | 12.599 |
| | TMTJ_part pos | .037 | 4.008 | 323.952 | 1.000 | -14.520 | 14.594 |
| | TMTJ_pos | 6.134 | 3.911 | 323.815 | 1.000 | -8.070 | 20.338 |
| DBLPN_neg | ASNB_neg | -4.068 | 2.543 | 322.426 | 1.000 | -13.304 | 5.169 |
| | ASNB_part pos | 2.900 | 2.902 | 323.810 | 1.000 | -7.641 | 13.442 |
| | ASNB_pos | -3.090 | 4.012 | 321.844 | 1.000 | -17.662 | 11.481 |
| | DBLPN_part pos | 3.337 | 2.487 | 323.760 | 1.000 | -5.694 | 12.369 |
| | DBLPN_pos | 4.769 | 2.262 | 323.951 | 1.000 | -3.447 | 12.986 |
| | Low6_neg | -3.508 | 2.237 | 304.634 | 1.000 | -11.638 | 4.621 |
| | Low6_part pos | 5.370 | 2.578 | 322.347 | 1.000 | -3.995 | 14.735 |
| | Low6_pos | 5.915 | 2.553 | 323.991 | 1.000 | -3.357 | 15.187 |
| | MTPJ_neg | -5.835 | 2.858 | 321.361 | 1.000 | -16.215 | 4.544 |
| | MTPJ_part pos | 7.737 | 2.802 | 301.940 | .934 | -2.447 | 17.921 |
| | MTPJ_pos | 2.918 | 3.050 | 314.197 | 1.000 | -8.164 | 14.001 |
| | other_neg | -3.499 | 2.376 | 323.877 | 1.000 | -12.127 | 5.129 |
| | other_part pos | 5.696 | 2.346 | 323.612 | 1.000 | -2.824 | 14.216 |
| | other_pos | 5.674 | 2.717 | 317.231 | 1.000 | -4.198 | 15.545 |
| | TMTJ_neg | -3.887 | 2.002 | 319.497 | 1.000 | -11.160 | 3.387 |
| | TMTJ_part pos | -3.053 | 2.542 | 315.860 | 1.000 | -12.290 | 6.184 |
| | TMTJ_pos | 3.043 | 2.374 | 322.503 | 1.000 | -5.580 | 11.667 |
| DBLPN_part pos | ASNB_neg | -7.405 | 2.468 | 320.858 | .444 | -16.369 | 1.559 |
| | ASNB_part pos | -.437 | 2.849 | 323.974 | 1.000 | -10.786 | 9.912 |
| | ASNB_pos | -6.428 | 3.973 | 322.584 | 1.000 | -20.859 | 8.003 |
| | DBLPN_neg | -3.337 | 2.487 | 323.760 | 1.000 | -12.369 | 5.694 |
| | DBLPN_pos | 1.432 | 2.181 | 323.965 | 1.000 | -6.490 | 9.354 |
| | Low6_neg | -6.846 | 2.185 | 321.280 | .289 | -14.783 | 1.091 |
| | Low6_part pos | 2.033 | 2.480 | 305.901 | 1.000 | -6.979 | 11.044 |
| | Low6_pos | 2.578 | 2.490 | 323.786 | 1.000 | -6.466 | 11.621 |

| | | | | | | | |
|---|---|---|---|---|---|---|---|
| | MTPJ_neg | -9.173 | 2.768 | 323.893 | .156 | -19.225 | .879 |
| | MTPJ_part pos | 4.400 | 2.746 | 294.779 | 1.000 | -5.584 | 14.383 |
| | MTPJ_pos | -.419 | 2.999 | 310.919 | 1.000 | -11.315 | 10.477 |
| | other_neg | -6.837 | 2.316 | 320.070 | .518 | -15.248 | 1.575 |
| | other_part pos | 2.359 | 2.274 | 323.948 | 1.000 | -5.900 | 10.617 |
| | other_pos | 2.336 | 2.674 | 303.983 | 1.000 | -7.381 | 12.053 |
| | TMTJ_neg | -7.224* | 1.933 | 323.946 | .034 | -14.244 | -.205 |
| | TMTJ_part pos | -6.391 | 2.467 | 318.072 | 1.000 | -15.351 | 2.570 |
| | TMTJ_pos | -.294 | 2.315 | 315.361 | 1.000 | -8.703 | 8.115 |
| DBLPN_pos | ASNB_neg | -8.837* | 2.240 | 315.620 | .015 | -16.974 | -.700 |
| | ASNB_part pos | -1.869 | 2.665 | 323.281 | 1.000 | -11.548 | 7.810 |
| | ASNB_pos | -7.860 | 3.836 | 321.930 | 1.000 | -21.794 | 6.074 |
| | DBLPN_neg | -4.769 | 2.262 | 323.951 | 1.000 | -12.986 | 3.447 |
| | DBLPN_part pos | -1.432 | 2.181 | 323.965 | 1.000 | -9.354 | 6.490 |
| | Low6_neg | -8.278* | 1.922 | 314.806 | .003 | -15.260 | -1.295 |
| | Low6_part pos | .601 | 2.285 | 317.415 | 1.000 | -7.699 | 8.901 |
| | Low6_pos | 1.146 | 2.257 | 323.519 | 1.000 | -7.052 | 9.344 |
| | MTPJ_neg | -10.605* | 2.605 | 319.207 | .009 | -20.067 | -1.142 |
| | MTPJ_part pos | 2.968 | 2.545 | 288.224 | 1.000 | -6.286 | 12.222 |
| | MTPJ_pos | -1.851 | 2.810 | 311.913 | 1.000 | -12.062 | 8.360 |
| | other_neg | -8.269* | 2.065 | 322.583 | .012 | -15.771 | -.766 |
| | other_part pos | .927 | 2.048 | 320.892 | 1.000 | -6.514 | 8.367 |
| | other_pos | .904 | 2.453 | 310.265 | 1.000 | -8.008 | 9.817 |
| | TMTJ_neg | -8.656* | 1.633 | 323.256 | .000 | -14.587 | -2.725 |
| | TMTJ_part pos | -7.822 | 2.231 | 322.566 | .079 | -15.926 | .281 |
| | TMTJ_pos | -1.726 | 2.077 | 307.979 | 1.000 | -9.275 | 5.824 |
| Low6_neg | ASNB_neg | -.559 | 2.263 | 320.067 | 1.000 | -8.781 | 7.662 |
| | ASNB_part pos | 6.408 | 2.672 | 323.892 | 1.000 | -3.297 | 16.114 |
| | ASNB_pos | .418 | 3.842 | 321.509 | 1.000 | -13.538 | 14.373 |

| | | | | | | | |
|---|---|---|---|---|---|---|---|
| | DBLPN_neg | 3.508 | 2.237 | 304.634 | 1.000 | -4.621 | 11.638 |
| | DBLPN_part pos | 6.846 | 2.185 | 321.280 | .289 | -1.091 | 14.783 |
| | DBLPN_pos | 8.278* | 1.922 | 314.806 | .003 | 1.295 | 15.260 |
| | Low6_part pos | 8.879* | 2.310 | 321.968 | .022 | .489 | 17.268 |
| | Low6_pos | 9.423* | 2.282 | 323.683 | .007 | 1.134 | 17.713 |
| | MTPJ_neg | -2.327 | 2.614 | 320.570 | 1.000 | -11.821 | 7.166 |
| | MTPJ_part pos | 11.246* | 2.537 | 306.860 | .002 | 2.025 | 20.466 |
| | MTPJ_pos | 6.427 | 2.824 | 310.698 | 1.000 | -3.834 | 16.687 |
| | other_neg | .009 | 2.081 | 323.000 | 1.000 | -7.549 | 7.566 |
| | other_part pos | 9.204* | 2.061 | 322.226 | .002 | 1.717 | 16.691 |
| | other_pos | 9.182* | 2.465 | 310.955 | .036 | .223 | 18.140 |
| | TMTJ_neg | -.379 | 1.641 | 315.998 | 1.000 | -6.340 | 5.583 |
| | TMTJ_part pos | .455 | 2.251 | 320.833 | 1.000 | -7.722 | 8.632 |
| | TMTJ_pos | 6.552 | 2.076 | 321.152 | .267 | -.988 | 14.091 |
| Low6_part pos | ASNB_neg | -9.438* | 2.561 | 312.436 | .041 | -18.743 | -.133 |
| | ASNB_part pos | -2.470 | 2.923 | 320.332 | 1.000 | -13.088 | 8.148 |
| | ASNB_pos | -8.461 | 4.034 | 320.975 | 1.000 | -23.114 | 6.192 |
| | DBLPN_neg | -5.370 | 2.578 | 322.347 | 1.000 | -14.735 | 3.995 |
| | DBLPN_part pos | -2.033 | 2.480 | 305.901 | 1.000 | -11.044 | 6.979 |
| | DBLPN_pos | -.601 | 2.285 | 317.415 | 1.000 | -8.901 | 7.699 |
| | Low6_neg | -8.879* | 2.310 | 321.968 | .022 | -17.268 | -.489 |
| | Low6_pos | .545 | 2.587 | 323.308 | 1.000 | -8.850 | 9.939 |
| | MTPJ_neg | -11.206* | 2.860 | 322.625 | .017 | -21.594 | -.817 |
| | MTPJ_part pos | 2.367 | 2.818 | 316.645 | 1.000 | -7.870 | 12.604 |
| | MTPJ_pos | -2.452 | 3.070 | 320.664 | 1.000 | -13.604 | 8.700 |
| | other_neg | -8.870* | 2.413 | 323.838 | .042 | -17.634 | -.105 |
| | other_part pos | .326 | 2.380 | 321.227 | 1.000 | -8.319 | 8.971 |
| | other_pos | .303 | 2.763 | 314.796 | 1.000 | -9.737 | 10.343 |
| | TMTJ_neg | -9.257* | 2.056 | 320.162 | .001 | -16.725 | -1.790 |

| | | | | | | | |
|---|---|---|---|---|---|---|---|
| | TMTJ_part pos | -8.423 | 2.559 | 323.966 | .169 | -17.718 | .871 |
| | TMTJ_pos | -2.327 | 2.423 | 321.342 | 1.000 | -11.126 | 6.473 |
| Low6_pos | ASNB_neg | -9.983* | 2.519 | 310.813 | .014 | -19.135 | -.830 |
| | ASNB_part pos | -3.015 | 2.911 | 323.973 | 1.000 | -13.589 | 7.559 |
| | ASNB_pos | -9.006 | 3.959 | 302.950 | 1.000 | -23.397 | 5.386 |
| | DBLPN_neg | -5.915 | 2.553 | 323.991 | 1.000 | -15.187 | 3.357 |
| | DBLPN_part pos | -2.578 | 2.490 | 323.786 | 1.000 | -11.621 | 6.466 |
| | DBLPN_pos | -1.146 | 2.257 | 323.519 | 1.000 | -9.344 | 7.052 |
| | Low6_neg | -9.423* | 2.282 | 323.683 | .007 | -17.713 | -1.134 |
| | Low6_part pos | -.545 | 2.587 | 323.308 | 1.000 | -9.939 | 8.850 |
| | MTPJ_neg | -11.750* | 2.850 | 323.489 | .007 | -22.100 | -1.400 |
| | MTPJ_part pos | 1.822 | 2.804 | 302.045 | 1.000 | -8.368 | 12.013 |
| | MTPJ_pos | -2.997 | 2.996 | 321.928 | 1.000 | -13.879 | 7.886 |
| | other_neg | -9.414* | 2.381 | 323.587 | .014 | -18.063 | -.766 |
| | other_part pos | -.219 | 2.360 | 323.789 | 1.000 | -8.789 | 8.351 |
| | other_pos | -.241 | 2.719 | 317.912 | 1.000 | -10.118 | 9.635 |
| | TMTJ_neg | -9.802* | 2.027 | 323.634 | .000 | -17.163 | -2.441 |
| | TMTJ_part pos | -8.968 | 2.540 | 318.355 | .073 | -18.196 | .260 |
| | TMTJ_pos | -2.872 | 2.396 | 312.208 | 1.000 | -11.579 | 5.835 |
| MTPJ_neg | ASNB_neg | 1.768 | 2.845 | 323.950 | 1.000 | -8.564 | 12.100 |
| | ASNB_part pos | 8.735 | 3.116 | 309.217 | .822 | -2.586 | 20.057 |
| | ASNB_pos | 2.745 | 4.214 | 323.653 | 1.000 | -12.561 | 18.051 |
| | DBLPN_neg | 5.835 | 2.858 | 321.361 | 1.000 | -4.544 | 16.215 |
| | DBLPN_part pos | 9.173 | 2.768 | 323.893 | .156 | -.879 | 19.225 |
| | DBLPN_pos | 10.605* | 2.605 | 319.207 | .009 | 1.142 | 20.067 |
| | Low6_neg | 2.327 | 2.614 | 320.570 | 1.000 | -7.166 | 11.821 |
| | Low6_part pos | 11.206* | 2.860 | 322.625 | .017 | .817 | 21.594 |
| | Low6_pos | 11.750* | 2.850 | 323.489 | .007 | 1.400 | 22.100 |
| | MTPJ_part pos | 13.573* | 3.086 | 295.607 | .002 | 2.353 | 24.792 |

| | | | | | | | |
|---|---|---|---|---|---|---|---|
| | MTPJ_pos | 8.754 | 3.310 | 311.353 | 1.000 | -3.274 | 20.782 |
| | other_neg | 2.336 | 2.671 | 323.931 | 1.000 | -7.366 | 12.038 |
| | other_part pos | 11.531* | 2.663 | 323.899 | .003 | 1.860 | 21.203 |
| | other_pos | 11.509* | 3.022 | 302.996 | .026 | .526 | 22.492 |
| | TMTJ_neg | 1.948 | 2.390 | 320.278 | 1.000 | -6.732 | 10.629 |
| | TMTJ_part pos | 2.782 | 2.795 | 323.525 | 1.000 | -7.371 | 12.935 |
| | TMTJ_pos | 8.879 | 2.713 | 309.334 | .181 | -.980 | 18.737 |
| MTPJ_part pos | ASNB_neg | -11.805* | 2.789 | 312.596 | .005 | -21.939 | -1.671 |
| | ASNB_part pos | -4.837 | 3.123 | 314.565 | 1.000 | -16.182 | 6.508 |
| | ASNB_pos | -10.828 | 4.177 | 323.107 | 1.000 | -26.001 | 4.345 |
| | DBLPN_neg | -7.737 | 2.802 | 301.940 | .934 | -17.921 | 2.447 |
| | DBLPN_part pos | -4.400 | 2.746 | 294.779 | 1.000 | -14.383 | 5.584 |
| | DBLPN_pos | -2.968 | 2.545 | 288.224 | 1.000 | -12.222 | 6.286 |
| | Low6_neg | -11.246* | 2.537 | 306.860 | .002 | -20.466 | -2.025 |
| | Low6_part pos | -2.367 | 2.818 | 316.645 | 1.000 | -12.604 | 7.870 |
| | Low6_pos | -1.822 | 2.804 | 302.045 | 1.000 | -12.013 | 8.368 |
| | MTPJ_neg | -13.573* | 3.086 | 295.607 | .002 | -24.792 | -2.353 |
| | MTPJ_pos | -4.819 | 3.239 | 307.229 | 1.000 | -16.590 | 6.952 |
| | other_neg | -11.237* | 2.655 | 282.299 | .005 | -20.896 | -1.577 |
| | other_part pos | -2.041 | 2.629 | 289.762 | 1.000 | -11.603 | 7.521 |
| | other_pos | -2.064 | 2.969 | 273.609 | 1.000 | -12.868 | 8.740 |
| | TMTJ_neg | -11.624* | 2.330 | 280.823 | .000 | -20.100 | -3.148 |
| | TMTJ_part pos | -10.790* | 2.796 | 276.668 | .022 | -20.966 | -.615 |
| | TMTJ_pos | -4.694 | 2.654 | 274.810 | 1.000 | -14.352 | 4.964 |
| MTPJ_pos | ASNB_neg | -6.986 | 3.024 | 323.597 | 1.000 | -17.968 | 3.996 |
| | ASNB_part pos | -.018 | 3.324 | 323.605 | 1.000 | -12.093 | 12.056 |
| | ASNB_pos | -6.009 | 4.345 | 323.998 | 1.000 | -21.790 | 9.772 |
| | DBLPN_neg | -2.918 | 3.050 | 314.197 | 1.000 | -14.001 | 8.164 |
| | DBLPN_part pos | .419 | 2.999 | 310.919 | 1.000 | -10.477 | 11.315 |

| | | | | | | | |
|---|---|---|---|---|---|---|---|
| | DBLPN_pos | 1.851 | 2.810 | 311.913 | 1.000 | -8.360 | 12.062 |
| | Low6_neg | -6.427 | 2.824 | 310.698 | 1.000 | -16.687 | 3.834 |
| | Low6_part pos | 2.452 | 3.070 | 320.664 | 1.000 | -8.700 | 13.604 |
| | Low6_pos | 2.997 | 2.996 | 321.928 | 1.000 | -7.886 | 13.879 |
| | MTPJ_neg | -8.754 | 3.310 | 311.353 | 1.000 | -20.782 | 3.274 |
| | MTPJ_part pos | 4.819 | 3.239 | 307.229 | 1.000 | -6.952 | 16.590 |
| | other_neg | -6.418 | 2.897 | 314.864 | 1.000 | -16.941 | 4.106 |
| | other_part pos | 2.778 | 2.856 | 322.610 | 1.000 | -7.594 | 13.150 |
| | other_pos | 2.755 | 3.203 | 295.757 | 1.000 | -8.890 | 14.401 |
| | TMTJ_neg | -6.805 | 2.632 | 300.384 | 1.000 | -16.371 | 2.761 |
| | TMTJ_part pos | -5.972 | 3.045 | 298.465 | 1.000 | -17.041 | 5.098 |
| | TMTJ_pos | .125 | 2.915 | 298.589 | 1.000 | -10.472 | 10.722 |
| other_neg | ASNB_neg | -.568 | 2.361 | 320.320 | 1.000 | -9.143 | 8.007 |
| | ASNB_part pos | 6.400 | 2.757 | 323.795 | 1.000 | -3.614 | 16.413 |
| | ASNB_pos | .409 | 3.898 | 320.985 | 1.000 | -13.751 | 14.568 |
| | DBLPN_neg | 3.499 | 2.376 | 323.877 | 1.000 | -5.129 | 12.127 |
| | DBLPN_part pos | 6.837 | 2.316 | 320.070 | .518 | -1.575 | 15.248 |
| | DBLPN_pos | 8.269* | 2.065 | 322.583 | .012 | .766 | 15.771 |
| | Low6_neg | -.009 | 2.081 | 323.000 | 1.000 | -7.566 | 7.549 |
| | Low6_part pos | 8.870* | 2.413 | 323.838 | .042 | .105 | 17.634 |
| | Low6_pos | 9.414* | 2.381 | 323.587 | .014 | .766 | 18.063 |
| | MTPJ_neg | -2.336 | 2.671 | 323.931 | 1.000 | -12.038 | 7.366 |
| | MTPJ_part pos | 11.237* | 2.655 | 282.299 | .005 | 1.577 | 20.896 |
| | MTPJ_pos | 6.418 | 2.897 | 314.864 | 1.000 | -4.106 | 16.941 |
| | other_part pos | 9.195* | 2.144 | 321.837 | .004 | 1.408 | 16.983 |
| | other_pos | 9.173 | 2.574 | 299.228 | .065 | -.184 | 18.531 |
| | TMTJ_neg | -.387 | 1.808 | 315.581 | 1.000 | -6.957 | 6.182 |
| | TMTJ_part pos | .446 | 2.375 | 304.828 | 1.000 | -8.184 | 9.077 |
| | TMTJ_pos | 6.543 | 2.211 | 300.047 | .510 | -1.495 | 14.581 |

| | | | | | | | |
|---|---|---|---|---|---|---|---|
| other_part pos | ASNB_neg | -9.764* | 2.343 | 321.708 | .006 | -18.273 | -1.254 |
| | ASNB_part pos | -2.796 | 2.717 | 320.610 | 1.000 | -12.665 | 7.074 |
| | ASNB_pos | -8.787 | 3.883 | 319.522 | 1.000 | -22.891 | 5.318 |
| | DBLPN_neg | -5.696 | 2.346 | 323.612 | 1.000 | -14.216 | 2.824 |
| | DBLPN_part pos | -2.359 | 2.274 | 323.948 | 1.000 | -10.617 | 5.900 |
| | DBLPN_pos | -.927 | 2.048 | 320.892 | 1.000 | -8.367 | 6.514 |
| | Low6_neg | -9.204* | 2.061 | 322.226 | .002 | -16.691 | -1.717 |
| | Low6_part pos | -.326 | 2.380 | 321.227 | 1.000 | -8.971 | 8.319 |
| | Low6_pos | .219 | 2.360 | 323.789 | 1.000 | -8.351 | 8.789 |
| | MTPJ_neg | -11.531* | 2.663 | 323.899 | .003 | -21.203 | -1.860 |
| | MTPJ_part pos | 2.041 | 2.629 | 289.762 | 1.000 | -7.521 | 11.603 |
| | MTPJ_pos | -2.778 | 2.856 | 322.610 | 1.000 | -13.150 | 7.594 |
| | other_neg | -9.195* | 2.144 | 321.837 | .004 | -16.983 | -1.408 |
| | other_pos | -.022 | 2.536 | 314.348 | 1.000 | -9.237 | 9.192 |
| | TMTJ_neg | -9.583* | 1.774 | 321.216 | .000 | -16.026 | -3.140 |
| | TMTJ_part pos | -8.749* | 2.349 | 308.286 | .036 | -17.286 | -.212 |
| | TMTJ_pos | -2.653 | 2.173 | 315.509 | 1.000 | -10.549 | 5.244 |
| other_pos | ASNB_neg | -9.741 | 2.731 | 313.393 | .064 | -19.664 | .181 |
| | ASNB_part pos | -2.774 | 3.072 | 313.038 | 1.000 | -13.934 | 8.387 |
| | ASNB_pos | -8.764 | 4.129 | 323.923 | 1.000 | -23.762 | 6.234 |
| | DBLPN_neg | -5.674 | 2.717 | 317.231 | 1.000 | -15.545 | 4.198 |
| | DBLPN_part pos | -2.336 | 2.674 | 303.983 | 1.000 | -12.053 | 7.381 |
| | DBLPN_pos | -.904 | 2.453 | 310.265 | 1.000 | -9.817 | 8.008 |
| | Low6_neg | -9.182* | 2.465 | 310.955 | .036 | -18.140 | -.223 |
| | Low6_part pos | -.303 | 2.763 | 314.796 | 1.000 | -10.343 | 9.737 |
| | Low6_pos | .241 | 2.719 | 317.912 | 1.000 | -9.635 | 10.118 |
| | MTPJ_neg | -11.509* | 3.022 | 302.996 | .026 | -22.492 | -.526 |
| | MTPJ_part pos | 2.064 | 2.969 | 273.609 | 1.000 | -8.740 | 12.868 |
| | MTPJ_pos | -2.755 | 3.203 | 295.757 | 1.000 | -14.401 | 8.890 |

| | | | | | | | |
|---|---|---|---|---|---|---|---|
| | other_neg | -9.173 | 2.574 | 299.228 | .065 | -18.531 | .184 |
| | other_part pos | .022 | 2.536 | 314.348 | 1.000 | -9.192 | 9.237 |
| | TMTJ_neg | -9.561* | 2.245 | 292.486 | .004 | -17.726 | -1.395 |
| | TMTJ_part pos | -8.727 | 2.725 | 287.163 | .232 | -18.637 | 1.184 |
| | TMTJ_pos | -2.630 | 2.579 | 286.355 | 1.000 | -12.009 | 6.749 |
| TMTJ_neg | ASNB_neg | -.181 | 2.001 | 313.602 | 1.000 | -7.449 | 7.088 |
| | ASNB_part pos | 6.787 | 2.467 | 322.534 | .959 | -2.173 | 15.747 |
| | ASNB_pos | .796 | 3.687 | 318.591 | 1.000 | -12.599 | 14.192 |
| | DBLPN_neg | 3.887 | 2.002 | 319.497 | 1.000 | -3.387 | 11.160 |
| | DBLPN_part pos | 7.224* | 1.933 | 323.946 | .034 | .205 | 14.244 |
| | DBLPN_pos | 8.656* | 1.633 | 323.256 | .000 | 2.725 | 14.587 |
| | Low6_neg | .379 | 1.641 | 315.998 | 1.000 | -5.583 | 6.340 |
| | Low6_part pos | 9.257* | 2.056 | 320.162 | .001 | 1.790 | 16.725 |
| | Low6_pos | 9.802* | 2.027 | 323.634 | .000 | 2.441 | 17.163 |
| | MTPJ_neg | -1.948 | 2.390 | 320.278 | 1.000 | -10.629 | 6.732 |
| | MTPJ_part pos | 11.624* | 2.330 | 280.823 | .000 | 3.148 | 20.100 |
| | MTPJ_pos | 6.805 | 2.632 | 300.384 | 1.000 | -2.761 | 16.371 |
| | other_neg | .387 | 1.808 | 315.581 | 1.000 | -6.182 | 6.957 |
| | other_part pos | 9.583* | 1.774 | 321.216 | .000 | 3.140 | 16.026 |
| | other_pos | 9.561* | 2.245 | 292.486 | .004 | 1.395 | 17.726 |
| | TMTJ_part pos | .834 | 2.009 | 305.758 | 1.000 | -6.468 | 8.136 |
| | TMTJ_pos | 6.930* | 1.819 | 291.046 | .026 | .317 | 13.543 |
| TMTJ_part pos | ASNB_neg | -1.014 | 2.538 | 320.424 | 1.000 | -10.233 | 8.204 |
| | ASNB_part pos | 5.953 | 2.897 | 320.750 | 1.000 | -4.569 | 16.476 |
| | ASNB_pos | -.037 | 4.008 | 323.952 | 1.000 | -14.594 | 14.520 |
| | DBLPN_neg | 3.053 | 2.542 | 315.860 | 1.000 | -6.184 | 12.290 |
| | DBLPN_part pos | 6.391 | 2.467 | 318.072 | 1.000 | -2.570 | 15.351 |
| | DBLPN_pos | 7.822 | 2.231 | 322.566 | .079 | -.281 | 15.926 |
| | Low6_neg | -.455 | 2.251 | 320.833 | 1.000 | -8.632 | 7.722 |

| | | | | | | | |
|---|---|---|---|---|---|---|---|
| | Low6_part pos | 8.423 | 2.559 | 323.966 | .169 | -.871 | 17.718 |
| | Low6_pos | 8.968 | 2.540 | 318.355 | .073 | -.260 | 18.196 |
| | MTPJ_neg | -2.782 | 2.795 | 323.525 | 1.000 | -12.935 | 7.371 |
| | MTPJ_part pos | 10.790* | 2.796 | 276.668 | .022 | .615 | 20.966 |
| | MTPJ_pos | 5.972 | 3.045 | 298.465 | 1.000 | -5.098 | 17.041 |
| | other_neg | -.446 | 2.375 | 304.828 | 1.000 | -9.077 | 8.184 |
| | other_part pos | 8.749* | 2.349 | 308.286 | .036 | .212 | 17.286 |
| | other_pos | 8.727 | 2.725 | 287.163 | .232 | -1.184 | 18.637 |
| | TMTJ_neg | -.834 | 2.009 | 305.758 | 1.000 | -8.136 | 6.468 |
| | TMTJ_pos | 6.097 | 2.379 | 291.160 | 1.000 | -2.553 | 14.746 |
| TMTJ_pos | ASNB_neg | -7.111 | 2.387 | 319.224 | .476 | -15.781 | 1.559 |
| | ASNB_part pos | -.143 | 2.746 | 324.000 | 1.000 | -10.118 | 9.832 |
| | ASNB_pos | -6.134 | 3.911 | 323.815 | 1.000 | -20.338 | 8.070 |
| | DBLPN_neg | -3.043 | 2.374 | 322.503 | 1.000 | -11.667 | 5.580 |
| | DBLPN_part pos | .294 | 2.315 | 315.361 | 1.000 | -8.115 | 8.703 |
| | DBLPN_pos | 1.726 | 2.077 | 307.979 | 1.000 | -5.824 | 9.275 |
| | Low6_neg | -6.552 | 2.076 | 321.152 | .267 | -14.091 | .988 |
| | Low6_part pos | 2.327 | 2.423 | 321.342 | 1.000 | -6.473 | 11.126 |
| | Low6_pos | 2.872 | 2.396 | 312.208 | 1.000 | -5.835 | 11.579 |
| | MTPJ_neg | -8.879 | 2.713 | 309.334 | .181 | -18.737 | .980 |
| | MTPJ_part pos | 4.694 | 2.654 | 274.810 | 1.000 | -4.964 | 14.352 |
| | MTPJ_pos | -.125 | 2.915 | 298.589 | 1.000 | -10.722 | 10.472 |
| | other_neg | -6.543 | 2.211 | 300.047 | .510 | -14.581 | 1.495 |
| | other_part pos | 2.653 | 2.173 | 315.509 | 1.000 | -5.244 | 10.549 |
| | other_pos | 2.630 | 2.579 | 286.355 | 1.000 | -6.749 | 12.009 |
| | TMTJ_neg | -6.930* | 1.819 | 291.046 | .026 | -13.543 | -.317 |
| | TMTJ_part pos | -6.097 | 2.379 | 291.160 | 1.000 | -14.746 | 2.553 |

Based on estimated marginal means

*. The mean difference is significant at the .05 level.

a. Dependent Variable: DPDup.

c. Adjustment for multiple comparisons: Bonferroni.
